# Effects of maternal obesity on Wharton’s Jelly mesenchymal stromal cells

**DOI:** 10.1038/s41598-017-18034-1

**Published:** 2017-12-14

**Authors:** Heba Badraiq, Aleksandra Cvoro, Antonio Galleu, Marisa Simon, Cristian Miere, Carl Hobbs, Reiner Schulz, Richard Siow, Francesco Dazzi, Dusko Ilic

**Affiliations:** 1 Stem Cell Laboratory, Assisted Conception Unit, School of Life Course Sciences, Department of Women and Children’s Health, Faculty of Life Sciences & Medicine, King’s College London, London, UK; 2 Genomic Medicine, Houston Methodist Research Institute, Houston, Texas USA; 3 Department of Haemato-Oncology, Rayne Institute, Faculty of Life Sciences & Medicine, King’s College London, London, UK; 4 Histology Laboratory, Wolfson Centre for Age-Related Diseases, Faculty of Life Sciences & Medicine, King’s College London, London, UK; 5 Department of Medical and Molecular Genetics, Division of Genetics and Molecular Medicine, King’s College London, London, UK; 6 Cardiovascular Division, Faculty of Life Sciences & Medicine, King’s College London, London, UK

## Abstract

We investigated whether maternal metabolic environment affects mesenchymal stromal/stem cells (MSCs) from umbilical cord’s Wharton’s Jelly (WJ) on a molecular level, and potentially render them unsuitable for clinical use in multiple recipients. In this pilot study on umbilical cords *post partum* from healthy non-obese (BMI = 19–25; n = 7) and obese (BMI ≥ 30; n = 7) donors undergoing elective Cesarean section, we found that WJ MSC from obese donors showed slower population doubling and a stronger immunosuppressive activity. Genome-wide DNA methylation of triple positive (CD73^+^CD90^+^CD105^+^) WJ MSCs found 67 genes with at least one CpG site where the methylation difference was ≥0.2 in four or more obese donors. Only one gene, *PNPLA7*, demonstrated significant difference on methylome, transcriptome and protein level. Although the number of analysed donors is limited, our data suggest that the altered metabolic environment related to excessive body weight might bear consequences on the WJ MSCs.

## Introduction

WJ-derived MSCs offer several advantages over those sourced from adult tissues. The material is easily obtained, the cells are more naive and primitive, they have a higher proliferation rate and expansion capability, whilst still maintaining strong immunomodulatory properties^1–4^. However, evidence suggest that WJ MSCs characteristics may be correlated with biological features of the neonates. MSCs from newborns that are small for gestational age have altered regulation of cell proliferation and oxidative stress^5^. Therefore, altered in utero metabolic environment might skew fetal MSC towards enhanced adipogenesis and fibrogenesis during intra-utero development and that may result in increased intramuscular fat and connective tissue^6^. In support of this hypothesis, sera of overweight people, reflecting their metabolic environment, promote *in vitro* adipocyte differentiation of bone marrow (BM) MSCs and amniotic (A) MSCs from obese women have a higher adipogenic potential^7,8^. Furthermore, the osteogenic response of undifferentiated BM MSCs to mechanical strain is inversely related to body mass index of the donor^9^, while the adipose-derived (AD) MSCs isolated from adipose tissue of obese patients have impaired proliferation, clonogenic ability and immune-phenotypes as well as a lower capacity for spontaneous or therapeutic repair than AD MSCs from non-obese, metabolically normal individuals^10,11^.

Animal studies have supported the observations in humans. A comparative study of AD MSCs isolated from Zucker diabetic fatty rats and their non-diabetic normal weight controls concluded that the impact of type 2 diabetes might compromise the efficiency of stem cell therapy^12^. BM MSCs isolated from the WNIN/GR-Obese rat model system designed to study obesity with Type 2 diabetes demonstrated a state of “disease memory”, with increased adipogenesis and non-responsiveness to high glucose^13^. A high-fat diet has been shown to increase interleukin (IL)-1, IL-6, and tumor necrosis factor (TNF)-α production by increasing nuclear factor (NF)-κB and attenuating peroxisome proliferator-activated receptor- (PPAR)-γ expression in BM MSCs of young Wistar rats^14^. Furthermore, diet-induced obesity altered the differentiation potential of the MSCs isolated from mouse bone marrow, adipose tissue and infrapatellar fat pad^15^.

The molecular mechanisms through which the altered intrauterine metabolic environment of a pregnant woman with excessive body weight might predispose offspring to long-term adiposity are unknown. Several recent studies suggested that epigenetic modifications could play a crucial role^16–20^. To evaluate whether the altered metabolic environment related to excessive body weight might bear consequences for the use of umbilical cord WJ MSCs in cellular therapy, we compared their growth, differentiation propensity into adipo-, chondro- and osteogenic lineages, immunomodulatory effect, genome-wide DNA methylation and transcriptome analyses in early passages of WJ MSCs isolated from healthy non-obese and obese donors (Figure S1).

## Results

### Growth and phenotypic profile of WJ MSCs from obese and non-obese donors show significant differences in population doubling (PD) time and CD56 expression

**Figure 1:**
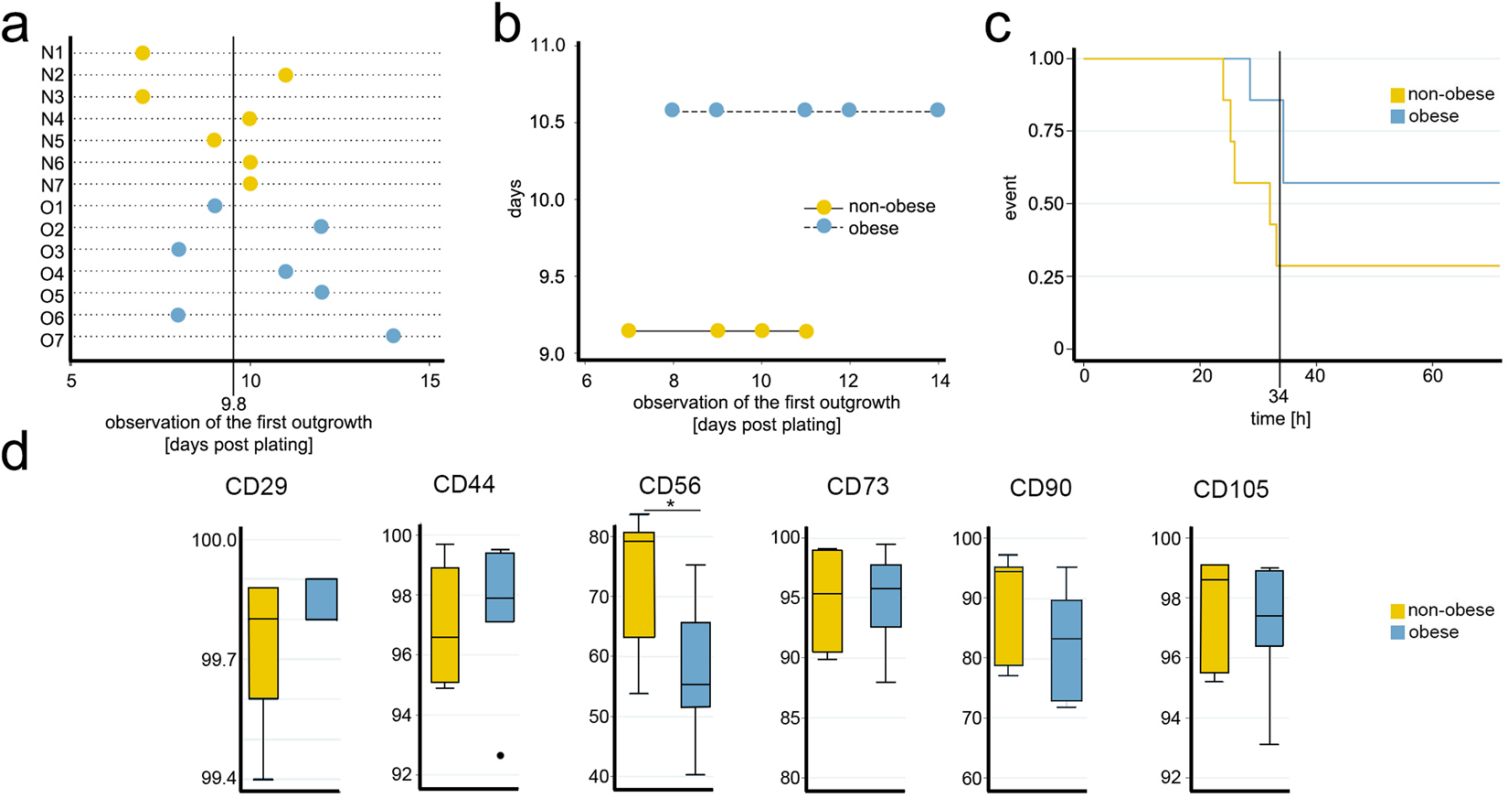
Significant differences between WJ MSCs isolated form non-obese and obese donors. (**a**) The WJ MSCs from the control groups showed an initial outgrowth on day 7 for donors N1 and N3 and on day 10 for donors N4, N6, and N7. Initial outgrowth for donors N5 and N2 was observed on days 9 and 11, respectively. In the cultures of explants from the obese group an initial outgrowth was evident on day 8 from donors O3 and O6 and on day 12 from donors O2 and O5. Donors O1 and O4 showed initial outgrowth on days 9 and 11, respectively. The explant culture from donor O7 was the slowest to establish and expand, showing the initial outgrowth on day 14. (**b**) Poisson model showed that the rate of initial outgrowth in the non-obese group (9.1 ± 1.5 days) was earlier than in the obese group (10.5 ± 2.2) but still not significant. The 95% confidence interval of the incidence rate ratio was 1.15 (0.827 to 1.61; p = 0.395). (**c**) The Kaplan-Meier curve using overall median of 34 h indicates that time to doubling of the WJ MSC from non-obese donors is superior. Stratified log-rank test for equality of functions has shown that the difference in the median time to doubling within the first 34 h is statistically significant (p = 0.048). (**d**) Expression profile of MSC surface markers. Mann-Whitney did not show significant difference between subpopulations of the two groups positive for CD29 (p = 0.268), CD44 (p = 0.482), CD73 (p = 0.949), CD90 (p = 0.249), and CD105 (p = 0.608), whereas CD56^+^ subpopulation was significantly smaller (p = 0.025) in the obese group (*p ≤ 0.05).

Initial outgrowth of WJ-MSCs from explants of the control group was evident on Day 7–11, whereas from the obese group on Day 8–14 (Fig. 1A). The mean (±standard deviation, SD) time to the first observation of outgrowth was 9.1 (±1.5) days in the non-obese donors and 10.5 (±2.2) days in the obese donors. However, the difference between the obese and non-obese groups in terms of the initial outgrowth of the cells was not significant by Mann-Whitney test (p = 0.220). Analysis based on the Poisson model showed that the timing of initial outgrowth in the non-obese group was earlier than in the obese group but was not significantly different (Fig. 1B).

The median (range) PD time of the WJ-MSCs between PD1 and PD2 was 32 (24.0 to 71.6) h in the non-obese group and 48 (28.5 to 96.0) h in the obese group. The overall median time to doubling was 34.2 (24.0 to 96.0) h. These results suggest that the population doubling time of cells from the obese group was somewhat longer than that of cells from the non-obese group; however, the difference was not significant per Mann-Whitney test (p = 0.084). The Kaplan-Meier log-rank test used to compare the cell doubling time in both groups in the first 34 h (overall median PD time) suggested that the difference in the time taken to double within the first 34 h between the groups was significant (p = 0.048); the PD time of the cells from the obese group was significantly longer than that of the cells from the non-obese group (Fig. 1C).

Although the majority of the cells in both groups were positive for all standard MSC markers (CD29, CD56, CD44, CD73, CD90 and CD105), Mann-Whitney testing of the expression profiles demonstrated significant difference between non-obese and obese donors in expression level of CD56, which showed significantly lower expression levels in cells from obese than non-obese donors (p = 0.025) (Fig. 1D). Both groups were negative for CD271 and MSCA-1 and for the haematopoietic cell surface markers CD45 and CD34 (data not shown). To avoid adding bias and to match the samples as closely as possible, all subsequent analyses were performed on the sorted triple positive (CD73^+^CD90^+^CD105^+^) WJ MSCs.

### WJ MSCs from obese and non-obese donors have a similar differentiation propensity

**Figure 2:**
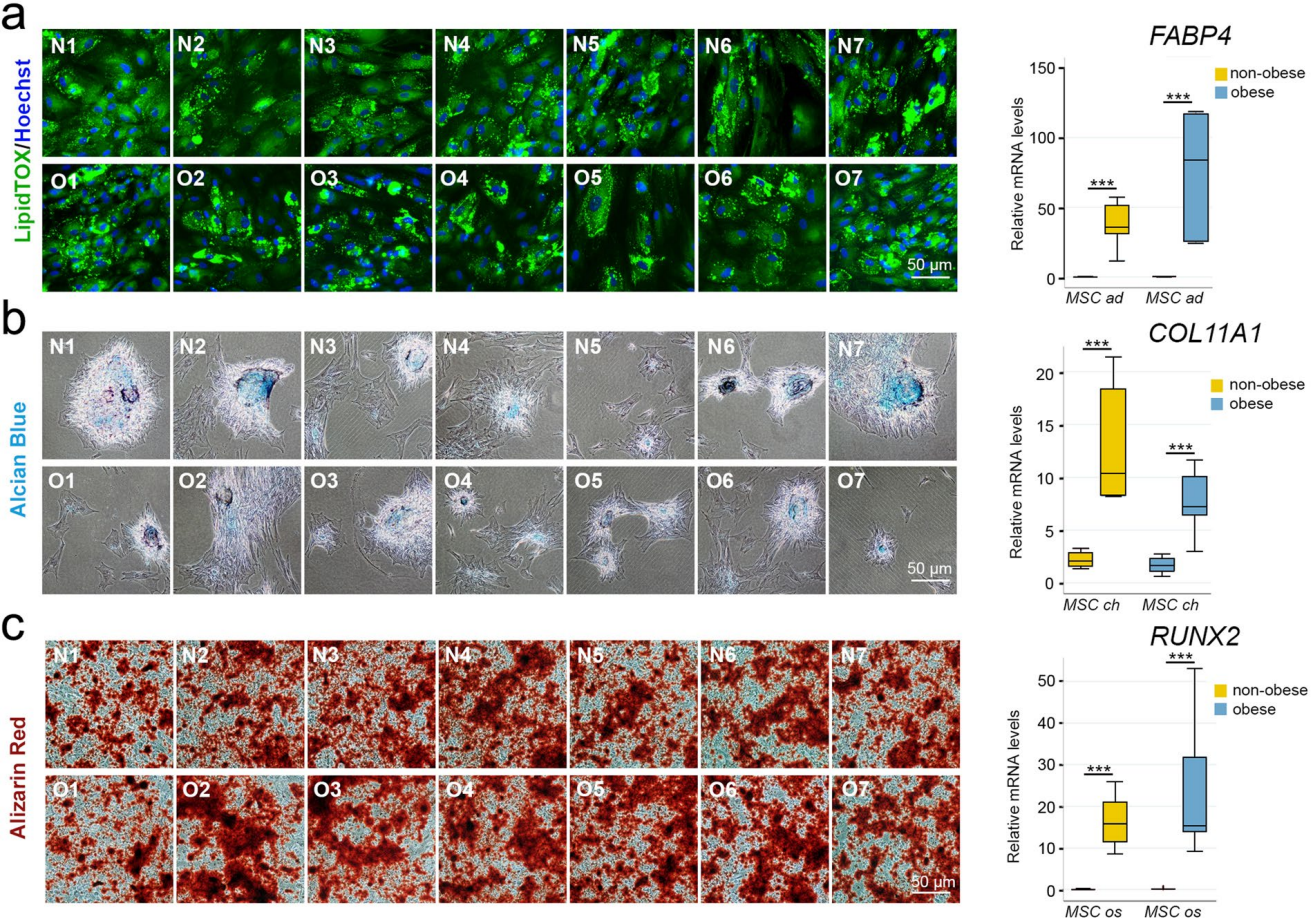
Propensity towards adipogenic, chondrogenic and osteogenic differentiation is similar between WJ MSCs from obese and non-obese donors. (**a**) Proadipogenic differentiation. Intracellular lipid droplets visualized with a fluorescent LipidTOX™ Green in WJ MSC from non-obese (N1–7) and obese donors (O1–7) following proadipogenic differentiation (left). Per Wilcoxon signed rank test, the expression level of adipogenic marker *FABP4*, as assessed by qPCR, was significantly higher in differentiated cells than in undifferentiated cells (***p ≤ 0.001) indicating adipogenesis (right). However, Mann-Whitney test did not find significant the overall differences between the obese and non-obese groups (p = 0.110). Analysis based on the Bootstrap model showed that the incidence rate ratio at the 95% confidence interval was −48.1 (−82.9 to 11.7). *ad*, cells undergoing proadipogenic differentiation; *MSC*, undifferentiated cells. (**b**) Prochondrogenic differentiation. Extracellular matrix rich in chondroitin sulfate glycosaminoglycans assessed with Alcian blue, pH 1.0 staining of WJ MSC from non-obese (N1–7) and obese (O1–7) donors following prochondrogenic differentiation (left). Per Wilcoxon signed rank test, the expression of chondrogenic marker *COLL11A1*, as assessed by qPCR, showed significant increase in WJ MSC from both non-obese and obese donors upon differentiation (***p ≤ 0.001), indicating chondrogenesis (right). However, Mann-Whitney test did not find significant differences between obese and non-obese groups (p = 0.063). Analysis based on the Bootstrap model showed that 95% confidence interval of the incidence rate ratio was 3.13 (−1.36 to 11.7). *ch*, cells undergoing prochondrogenic differentiation; *MSC*, undifferentiated cells. (**c**) Proosteogenic differentiation. Extracellular calcium deposits stained with Alizarin Red in WJ MSC from non-obese (N1–7) and obese (O1–7) donors following proosteogenic differentiation (left). Expression of osteogenic marker *RUNX2* assessed by qPCR showed significant increase in WJ MSC from both non-obese and obese donors upon differentiation (***p ≤ 0.001) indicating osteogenesis (right). However, Mann-Whitney test did not find differences between the groups significant (p = 0.482). Analysis based on the Bootstrap model showed that the 95% confidence interval of the incidence rate ratio was 0.54 (−15.4 to 10.4). *MSC*, undifferentiated cells; *os*, cells undergoing proosteogenic differentiation.

Next, we assessed adipo-, chondro- and osteogenic differentiation potential of triple positive (CD73^+^CD90^+^CD105^+^) WJ MSCs from the two groups (Fig. 2).

Lipid droplets were positively stained with LipidTOX dye in all samples after two weeks of exposure to proadipogenic medium (Figs 2A and S2). Per Wilcoxon signed rank test, mRNA expression level (mean ± SD) of the adipogenic marker fatty acid binding protein 4 (*FABP4*) was significantly higher in differentiated cells (56.5 ± 34.7) than in undifferentiated cells 0.23 ± 0.12 (***p = 0.001), confirming adipogenesis. Mann-Whitney test did not find significant the overall differences between the obese and non-obese groups (p = 0.110).

Similarly, following two weeks of exposure to chondrogenic medium, cells from all donors were stained positively with Alcian blue and chondrogenic marker collagen type XI alpha 1 chain (*COL11A1*) expression (Fig. 2B). Per Wilcoxon signed rank test, the mRNA expression level of *COL11A1* (mean ± SD) was significantly higher in differentiated (10.3 ± 4.94) than in undifferentiated cells 1.92 ± 0.74 (***p = 0.001), indicating the chondrogenesis. Mann-Whitney test did not find significant differences between obese and non-obese groups (p = 0.063).

Finally, we assessed osteogenic potential with Alizarin red staining and measuring mRNA levels of runt related transcription factor 2 (*RUNX2*), osteogenic-specific marker (Fig. 2C). The significant differences between the differentiated 19.7 ± 11.6 and undifferentiated cells 0.10 ± 0.09 (***, p = 0.001) per Wilcoxon signed rank test indicated osteogenesis. Mann-Whitney test did not find differences between the groups significant (p = 0.482).

### WJ MSCs from obese donors exhibit a stronger immunomodulatory potential than those from non-obese donors

**Figure 3:**
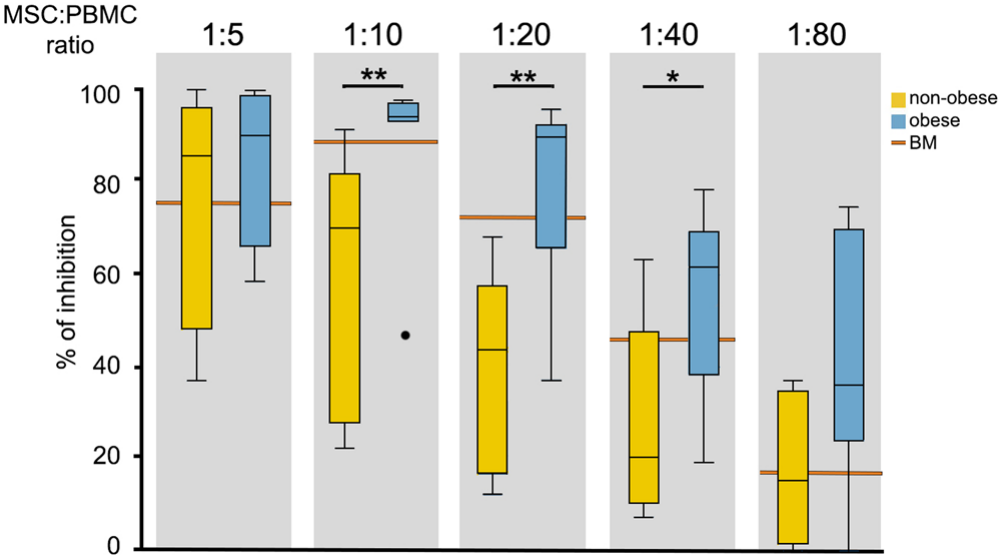
WJ MSCs from obese donor show significantly higher immunomodulatory potential than the cells from non-obese donors. WJ MSC from obese donors (n = 7) inhibited phytohemagglutinin-stimulated human PBMC proliferation in dose-dependent manner in a similar fashion as the BM MSC. The data were analysed using Two-sample Wilcoxon rank-sum (Mann-Whitney) test. Confidence interval for median difference was calculated using Bootstrap method. Immunomodulatory potential of the cells from non-obese donors (n = 7) was significantly lower at 1:10 (**p ≤ 0.01), 1:20 (**p ≤ 0.01) and 1:40 (*p ≤ 0.05) MSC:PBMC ratios.

Since MSC have the ability of exerting potent immunosuppressive and immunoregulatory effects^21–23^, we investigated whether the altered metabolic environment of the obese pregnancies affects WJ MSC immunomodulatory properties. Triple positive (CD73^+^CD90^+^CD105^+^) WJ MSCs from both non-obese (n = 7) and obese (n = 7) donors were compared for their ability to inhibit phytohemagglutinin A (PHA)-induced proliferation of peripheral blood mononuclear cells (PBMC). The data were analysed using Two-sample Wilcoxon rank-sum (Mann-Whitney) test. Confidence interval for median difference was calculated using Bootstrap method. A significant difference was observed when the cells were cultured in 1:10, 1:20 and 1:40 ratio, but not 1:5 or 1:80 suggesting that WJ MSC from obese donors were more immunosuppressive than those from normal controls and paralleled the immunomodulatory capabilities of BM MSCs (Fig. 3).

### Effect of maternal obesity on the DNA methylation profile of WJ MSCs

To investigate whether differences in cell proliferation, subpopulation profile and immunomodulatory potential reflect differences in epigenetic regulation of gene expression due to altered metabolic environment, we analysed whole epigenome DNA methylation using Illumina’s 450k array.

**Figure 4:**
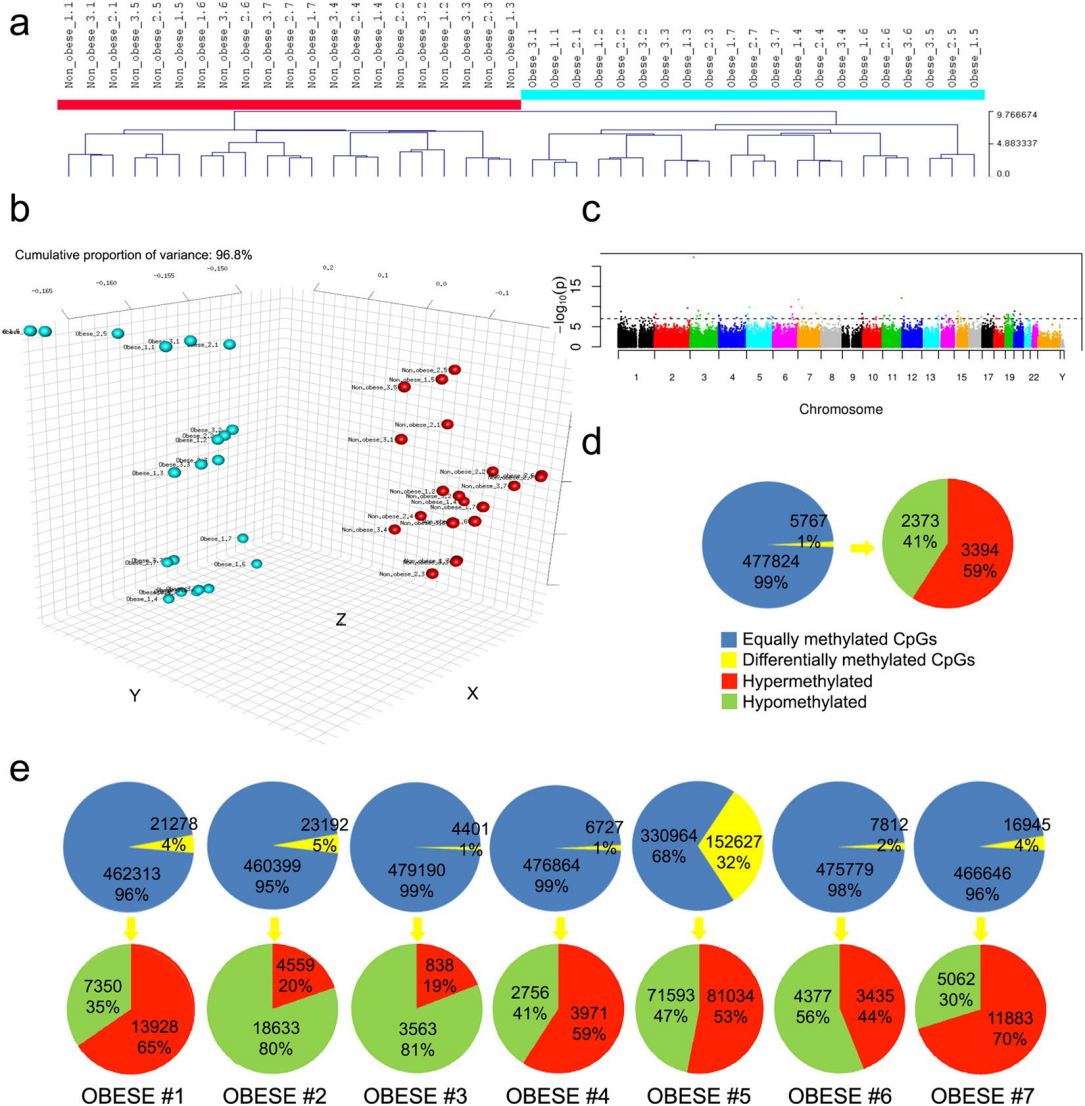
DNA methylation portrait. (**a**) Hierarchical clustering analysis based on epigenome-wide DNA methylation shows that the WJ MSC isolated from non-obese (n = 7) and obese donors (n = 7) cluster separately. WJ SMC from each donor were analysed in triplicates. (**b**) Principal component analysis (PCA) demonstrated that the WJ MSC from non-obese and obese donors formed separate clusters. (**c**) Manhattan plot indicates that altered methylation state was spread across all chromosomes. (**d**) Graphic showing percentage of differentially methylated CpG sites between averages of non-obese (n = 7) and obese (n = 7) samples. (**e**) Comparative analyses of WJ MSC from each obese donor vs. an average from all non-obese donors. Statistically significant alteration of methylation state was seen in 1–5% of all CpG sites in 6 out of 7 donors, whereas 32% of CpG sites were different in the samples from obese donor #5.

On average, the arrays passed the quality control thresholds (Figure S3). Given that there are three technical replicate arrays per sample, it would have been feasible to exclude the arrays with poorer performance without a reduction in the number of represented donors. However, given the hierarchical clustering result (triplicates of technical replicates always immediately clustered together), the inclusion of the arrays with poorer performance does not seem to have had much of an effect on the analysis. Hierarchical clustering of the arrays resulted in clearly distinct clusters of WJ MSCs isolated from samples of non-obese versus obese donors (Fig. 4A,B), while the technical triplicates for each sample were immediately clustered together. A Manhattan plot displaying the significance of the associations by chromosomes indicated that the altered methylation state was distributed across all chromosomes (Fig. 4C). Chromosome 1 harboured most of the differentially methylated CpGs, and Chromosome Y the fewest. The majority of the differentially methylated CpGs were either located in gene bodies (38% of hypermethylated and 31% of hypomethylated) or were intergenic (29% of hypermethylated and 34% of hypomethylated) (Figure S4). Based on the CpG content of the local genomic neighbourhood, the differentially methylated CpGs were isolated CpGs in regions defined as “open sea regions” (43% of hypermethylated and 47% of hypomethylated).

Comparison of the average of all non-obese with the average of all obese samples showed that about 1% of the assayed CpG sites (5767) were significantly differentially methylated (≥20% difference in methylation). Of these sites, 41% (2373) were hypomethylated and 59% (3394) were hypermethylated (Fig. 4D). We then compared DNA methylation between each individual obese donor and the group of non-obese donors (Fig. 4E). The differences in terms of the fraction of differentially methylated CpGs ranged from 1% (donors #3 and #4) to 32% (donor #5). Among CpG sites with significantly different methylation, hypomethylation was predominant in three donors (donor #2: 80%, donor #3: 81% and donor #6: 56%), while hypermethylation was more frequent in four donors (donor #1: 65%, donor #4: 59%, donor #5: 53% and donor #7: 70%). The statistical power for detecting a 20% methylation difference in our data set of 21 versus 21 samples (7 non-obese versus 7 obese donors, each in triplicate; Fig. 4A–C) was around 99%. The same was true of the 3 versus 21 samples comparison (one obese versus 7 non-obese donors, each in triplicate; Fig. 4D). Although the number of samples analysed is limited, our data suggest that maternal obesity might have a lasting effect on the DNA methylation profile of WJ MSCs isolated from umbilical cord explants and cultured *in vitro* for at least one passage.

Next, we looked for CpG sites where DNA methylation might be influenced by the altered metabolic environment of obese donors. The criterion was that the particular CpG site had to be significantly differentially methylated in four or more donors. We observed such CpG sites in 67 genes (Figure S4). Six out of seven obese donors shared 3 genes (*NTM*, *SIM2* and *STT3A*), five out of seven shared 22 genes and four out of seven shared 42 genes. However, among all these genes, only three had more than one differentially methylated CpG: *MAD1L1* (2), *TRIM10* (4) and *ZNF714* (5). The CpGs in *MAD1L1* and *TRIM10* were significantly differentially methylated in four donors, and *ZNF714* in five donors.

**Figure 5:**
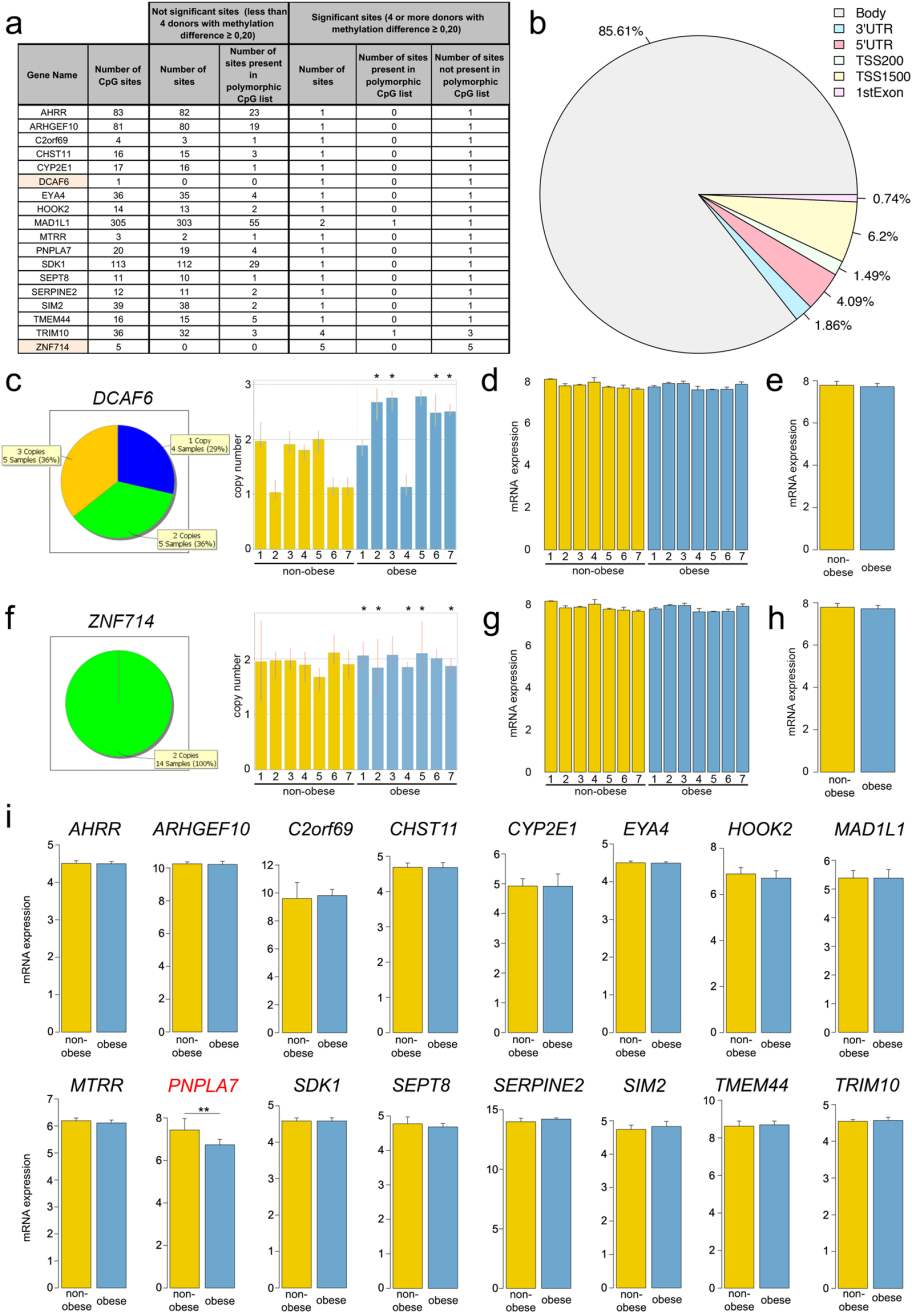
Genes with significantly different DNA methylation in one or more CpG sites in non-polymorphic regions. (**a**) List of 18 genes that have one of more CpG sites in non-polymorphic regions with difference in methylation ≥20%. *DCAF6* and *ZNF714*, highlighted orange, have variable copy number in genome. (**b**) Proportion of the locations of CpG sites in the 18 genes. TSS, transcription start site; TSS1500, the sequence region from −200 to −1,500 nucleotides upstream of the transcription start site; TSS200, the region from −200 nucleotides upstream to the TSS itself; UTR, untranslated region. (**c**) TaqMan Copy Number Assays for *DCAF6* showed that among 14 donors, four have one copy of the gene (N2, N6, N7 and O4), five have two copies (N1, N3, N4, N5, and O1) and also five have three copies (O2, O3, O5, O6, and O7). All four WJ MSC samples that showed significantly different *DCAF6* DNA methylation (*) originated from the donors that have three copies of the gene (O2, O3, O6, and O7). Each sample bar represents the mean calculated copy number and error bars show the standard deviation for three replicates. (**d** and **e**) Expression gene array did not detect significant difference *DCAF6* mRNA expression in any of the samples indicating that duplicated *DCAF6* genes might not be functional. (**f**) TaqMan Copy Number Assays for *ZNF714* showed that all non-obese and obese donors have two copies of the *ZNF714* gene. *Five donors that had significantly different DNA methylation of all CpG sites in *ZNF714* (O1, O2, O4, O5, and O7). Each sample bar represents the mean calculated copy number and error bars show the standard deviation for three replicates. (**g** and **h**) Expression gene array did not detect significant difference *ZNF714* mRNA expression in any of the samples indicating that the extra copy of *ZNF714* gene might be either functional or not in all donors uniformly. (**i**) The mRNA expression levels of 16 genes showed no differences in the obese and non-obese groups. The Y axis represents the log2 of the normalized intensity values. The single exception was the *PNPLA7* gene, which showed higher expression in the non-obese group than in the obese group (**p ≤ 0.01), the adjusted p-values from Linear Models for Microarray and RNA-Seq Data (limma) statistical test^57,58^.

The genes were further filtered based on a list of polymorphic CpG sites^24^ and segmental duplications^25^. In 18 out of the 67 genes with differentially methylated CpGs, the CpG sites were in non-polymorphic regions (Fig. 5A). However, two genes (*DCAF6* and *ZNF714*) resided in segmentally duplicated regions. Most of the differentially methylated CpGs were located in the gene body (85.61%), whereas promoter areas (TSS1500, TSS200 and 5′UTR) and the first exon accounted together for 12.53% of sites (Fig. 5B).

Copy number assays analysis of ligand-dependent coactivator of nuclear receptors *DDB1* and *CUL4* Associated Factor 6 (*DCAF6*) revealed that donors have variable copy number of the gene: four donors had 1 copy, five donors 2 and five donors 3 copies (Fig. 5C). All donors that displayed significant difference in *DCAF6* methylation had three copies of the gene (asterisks), which can explain a difference detected in the methylation array. Indeed, *DCAF6* mRNA expression levels did not show any significant difference among individual donors (Fig. 5D) or two groups (Fig. 5E). Similar analysis found that all 14 donors have two copies of the zinc finger protein 714 (*ZNF714*) gene, including those five that have all five CpG sites differently methylated (asterisks) (Fig. 5F). We could not detect a difference in mRNA levels among individual donors (Fig. 5G) or two groups (Fig. 5H), which suggest that in our case the methylation of these sites per se did not affect gene expression.

To see whether differences in methylation of the CpG sites led to different levels of gene expression, we used a whole transcriptome array. We found 244 regulated genes with fold-change ≥ 1.5 and uncorrected p-value ≤ 0.05 (Figure S5). Six out of seven samples from obese donors clustered together, whereas one (donor O3) clustered with non-obese (Figure S5). According to the Kyoto Encyclopedia of Genes and Genomes (KEGG) database (www.genome.jp/kegg/), putative pathways involved are metabolism by cytochrome P450 (6.7% genes), extracellular matrix-receptor interaction (6.0%) and hematopoietic cell lineage (4.7%), whereas REACTOME database (www.reactome.org) suggested involvement of mitotic cell cycle (3.9%) and metabolism of proteins (1.8%) pathways (Figure S6). Gene Ontology (GO) analysis (www.geneontology.org) found that among the ten the most significant biological processes involved, nine are linked to cell cycle, which might explain our finding that the cells from non-obese donors proliferate somewhat faster (Figs 1C and S6). Among the six the most significant cellular components involved, five are directly or indirectly linked to extracellular matrix signalling, which matches KEGG database (Figure S6).

Among the remaining 16 genes with significantly different methylation of CpG sites between two groups, only one, Patatin-like Phospholipase Domain containing Protein A 7(*PNPLA7*), had significantly different mRNA expression levels between non-obese and obese groups (Fig. 5I).

### PNPLA7 is downregulated in the samples from obese donors

**Figure 6:**
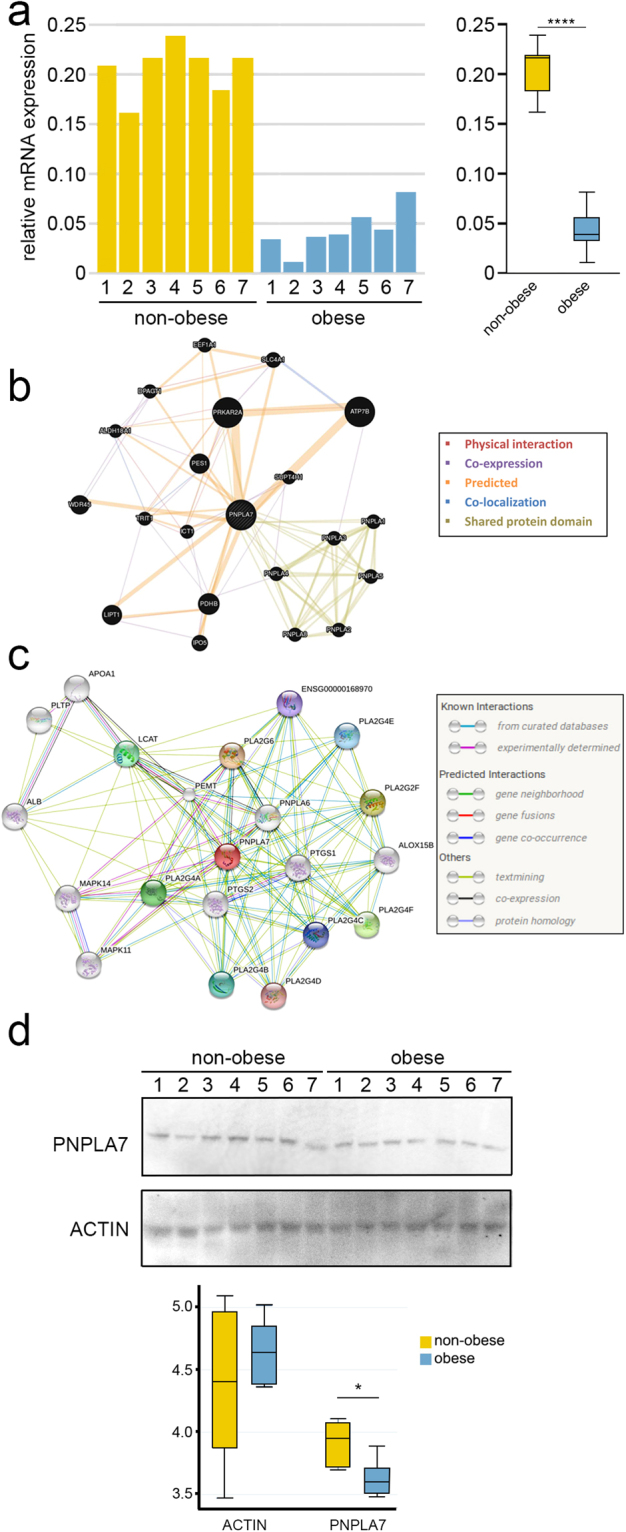
PNPLA7 expression is lower in the WJ MSC from obese donors. (**a**) The transcriptome array data were validated using *PNPLA7*-specific qPCR. Mann-Whitney test showed that there is indeed a significant difference between the two groups (****p ≤ 0.0001). The mean ± SD of the gene expression level was 20.6 ± 2.55 in the non-obese group and 4.3 ± 2.16 in the obese group. Analysis based on the Bootstrap model showed that the 95% confidence interval of the incidence rate ratio was 0.18 (0.15 to 0.21). (**b**) The GeneMANIA Cytoscape plugin *PNPLA7* gene function prediction using association approach. (**c**) Search Tool for the Retrieval of Interacting Genes/Proteins (STRING) predicted protein–protein interactions for PNPLA7. (**d**) Mann-Whitney test of the Western blot data indicates that the difference between the two groups is also maintained at the protein level (*p ≤ 0.01).

We confirmed the transcriptome array data with *PNPLA7*-specific qPCR (Fig. 6A). Mann-Whitney test confirmed that there is indeed a significant difference between the two groups (p = 0.001). The mean (±SD) of the gene expression level was 20.6 (±2.55) in the non-obese group and 4.3 (±2.16) in the obese group. Analysis based on the Bootstrap model showed that the 95% confidence interval of the incidence rate ratio was 0.18 (0.15 to 0.21).

To detect possible known signalling cascades and interaction between *PNPLA7* and other genes, we searched the interaction networks using GeneMANIA (Fig. 6B) and String V10.0 (Fig. 6C). According to GeneMANIA, *PNPLA7* has predicted interaction with 14 genes including *PRKAR2A* and *ATP7B*, whereas *PNPLA7* has experimentally determined interactions with *MAPK11*, *MAPK14* and *PEMT* according to String. However, we did not find any significant difference in gene expression levels in any of the genes highlighted by either of the two programmes.

PNPLA7 is integral component of lysosomal, mitochondrial, nuclear and endoplasmatic reticulum membrane and it is involved in metabolic and developmental processes. Western blot analysis indicated that the difference between the obese and non-obese groups is maintained on the protein level (Figs 6D and S7). The mean (±SD) value of PNPLA7 was lower 0.36 ± 0.01 in the obese group than in the non-obese group 0.39 ± 0.01. Per Mann-Whitney test, the data show that significant difference at the protein level was present between the obese and non-obese groups (p = 0.006). Actin, used as an essay control, showed no significant difference between the obese and non-obese group.

## Discussion

Epigenetic alterations that modulate gene expression as consequence of stressful conditions during prenatal development have been convincingly demonstrated in numerous animal and human studies (reviewed in refs^17,26–29^). The altered metabolic environment *in utero* due to maternal obesity could be considered stressful to the developing foetus, and indeed, maternal obesity is associated with differential expression of multiple genes^30^. Although the number of samples we analysed was limited and, power was therefore insufficient to detect small but significant differences, we were still able to detect significant differences between non-obese and obese pregnancies in cell proliferation, immunomodulatory properties, DNA methylation and gene expression of WJ MSCs. In addition, we cannot exclude the possibility that some of the effects produced by the altered *in utero* environment were lost during *in vitro* expansion.

A study demonstrated that specific changes in global methylation in the placenta tissue, but not in the umbilical cord blood, were significantly associated with maternal gestational diabetes mellitus (GDM), preeclampsia and obesity during pregnancy^31^. These findings suggest that the association between maternal obesity and changes in DNA methylation may be tissue-specific. No other study has investigated an association between maternal obesity and DNA methylation in WJ MSCs. The majority studies in this area have used either cord blood or placental samples, often looking for alteration in the methylation of specific genes.

Analysing offspring cord blood, Liu *et al*.^32^ have shown that maternal preconception BMI might lead to alterations in offspring DNA methylation in genes relevant to the development of a range of complex chronic diseases. Another study^33^ found that in cord blood, aryl-hydrocarbon receptor repressor (*AHRR*) DNA methylation was 2.1% higher in offspring of obese versus normal weight mothers. In our cohort four out of seven obese donors had more than >20% higher methylation of one CpG site in *AHRR*, though there was no difference in *AHRR* mRNA levels (Fig. 5i).

Similarly, studies have indicated that obesity during pregnancy affects the DNA methylation of the leptin (*LEP*) promoter region in placental tissue^34^, whereas in a mouse model was shown that maternal obesity and diabetes cause widespread epigenetic changes and alter hepatic gene expression in male offspring^35^. Another study, however, found that maternal GDM is associated with genome-wide DNA methylation changes in both the placenta and the cord blood of the exposed offspring^36^, contradicting the notion of a tissue-specific association^37^. The differences between the studies could arise from the use of different methods to assess DNA methylation. For example, methylated DNA immunoprecipitation provides coverage of the whole genome but is limited in resolution, while reduced representation bisulfite sequencing and Nimblegen MethSeq provide single CpG resolution but limited to CpG islands and promoters^38^.

It should be noted that the differential methylation analysis could be confounded by ethnicity. However, in our case, obese donors that showed a significant difference in *PNPLA7* methylation were mixed: two of them were Caucasians (O2, O3), one African Black (O4) and one Caribbean Black (O7), suggesting that the observed difference was not linked to ethnicity.

An interesting finding of our study was that the WJ MSCs from obese donors have a significantly higher immunosuppressive potential than their counterparts from non-obese donors. Obesity is, in general, accompanied by metaflammation, i.e., low-grade and chronic inflammation orchestrated by metabolic cells in response to excess nutrients and energy^39,40^. It is widely accepted that the MSCs’ immunosuppressive capability is not constitutive but results from the exposure to an inflammatory microenvironment^41^. The inflammatory cytokines and chemokines that likely are elevated in response to the altered metabolic status of obese donors, for example, TNF-α, may stimulate the WJ MSCs from obese pregnancies to secret higher than normal levels of various immunomodulatory factors and therefore, have a stronger inhibitory effect on the proliferation of PHA-stimulated PBMCs.

A couple of studies investigating correlation between adipogenic potential of WJ MSC and measures of infant body composition at birth found that MSC from infants born to obese mothers exhibit greater potential for adipogenesis^31,42^. This was linked to altered GSK-3β/β-catenin signalling in MSCs of infants exposed to maternal obesity. We, however, did not find a significant difference in differentiation potential. Several factors can contribute to such discrepancy in results: i) To investigate differentiation, we used triple positive CD73^+^CD90^+^CD105^+^ WJ MSCs, whereas they used mixed population, ii) Adipogenesis has been induced in a different way; in our experiment, the cells were exposed to pro-adipogenic medium continuously for 14 days, whereas in the other study the cells were cultured for 21 days with the three cycles of adipogenic induction medium and the maintenance medium in between^31^.

Our study singled out PNPLA7, an insulin- and glucose-regulated lysophospholipase that plays a putative role in reducing cellular lysophosphatidic acid (LPA; 1-acyl-*sn*-glycerol-3-phosphate) under fasting, when lipid stores are mobilised for energy production^43–45^. LPA is upregulated with adipocyte differentiation and obesity^46^ and, therefore, it does not come as a surprise that *PNPLA7* would be then less expressed in samples from obese pregnancies. In addition, it has also been reported that DNA methylation regulates *PNPLA7* expression - it is down-regulated in hepatocellular carcinoma cell lines and tissue samples, via the mechanism of transcriptional silencing by promoter hypermethylation^47^.

PNPLA7 is ubiquitously expressed transmembrane protein associated with lysosomes, mitochondria, and endoplasmic reticulum as well as microtubule organizing center^48^. However, our knowledge about PNPLA7 is still quite limited. Resolving specific role of PNPLA7 expression and activity in metabolic pathways associated with obesity will require significant additional work, the most importantly gain- and loss-of-function studies *in vitro* and *in vivo*. Against the background of the published data, our pilot study suggested that if significant differences in the *PNPLA7* DNA methylation or expression could be correlated with onset of obesity in childhood, PNPLA7 expression might be used as a potential biomarker. Whereas a correlation between body weight and PNPLA7 expression is general or restricted only to WJ MSC remains to be seen.

Although the number of analysed donors is limited, our data suggest that the effects of maternal obesity on WJ MSCs isolated from umbilical cord explants related to use of these cells for allogeneic therapy are relatively restricted and a concern that the abnormal metabolic environment related to excessive body weight might bear consequences for the use of umbilical cord MSCs in cellular therapy may not be warranted.

## Experimental section

### Human samples

Collection of the samples was described previously^49^. Briefly, following informed consent, for this study we recruited seven healthy obese pregnant women with BMI ≥ 30 and seven healthy non-obese with BMI between 21 and 25 at the gestational age of 37 weeks (Figure S1). All non-obese women were Caucasians aged between 35 and 43, whereas among the obese group, three were Caucasians (aged 34, 35 and 39) and four were Black, two African (aged 34 and 38) and two Caribbean (aged 28 and 35). All deliveries were elective Caesarean sections without complications and babies were healthy with no apparent abnormalities.

After inspection, the UC was cut, washed and transported to the laboratory in PBS, supplemented with antibiotics and antimycotics (Life Technologies). The UK National Health Service Research Ethics Committee approved the protocol (12/NE/0371). Clinical grade BM MSCs used as a control in some of the experiments were distributed for compassionate use under the hospital exemption scheme (EC No 1394/2007). All methods were performed in accordance with the relevant guidelines and regulations. Informed consent was obtained from all subjects and the experiments conformed to the principles set out in the WMA Declaration of Helsinki and the NIH Belmont Report. No financial inducements are offered for donation.

### Derivation of MSCs from UC explants and cell culture

The derivation of MSCs from UC explants and standard cell culture were described in detail previously^1,49^. Briefly, the 5–10 mm^2^ explants were cultured in DMEM supplemented with 10% fetal calf serum (Hyclone) at 37 °C, 5% CO_2_, 5% O_2_. The first harvest from explants is termed passage (P) 0 and following dissociation with TrypLE™ Select (Life Technologies), the cells, 7000/cm^2^, were re-plated under the same conditions. For DNA methylation analyses, we used the cells from P1 exclusively, and for all other experiments, we used the cells from both P1 and P2.

### Flow cytometry analyses

Flow cytometry analyses were performed as described^1^. Acute myeloid leukemia cell line Katsumi was used as a CD34^+^CD45^+^ control. Antibodies were purchased either form Miltenyi or BD Biosciences.

### Differentiation assays

We assessed the differentiation potential of WJ MSCs in three independent experiments. Upon plating into 4-well dishes, the cells were cultured first under standard conditions for 2 days. Medium was then replaced with supplemented complete STEMPRO® adipogenesis, chondrogenesis or osteogenesis differentiating medium (Gibco®, Life Technologies), and replaced on a regular basis for up to three weeks. The cells were then fixed with 4% paraformaldehyde/PBS for 30 min.

After 21-day incubation under differentiation conditions, the cells were washed with PBS and fixed with 4% formaldehyde solution for 30 minutes at room temperature (Sigma-Aldrich). Following fixation, the cells were washed twice with PBS and incubated for 30 minutes in a 1:100 dilution of LipidTOX^TM^ Green Neutral Lipid Stain (Invitrogen), which detects cellular lipids. The cells were washed again twice with PBS and mounted in Vectashield Mounting Medium supplemented with 1.5 μg/ml 4′,6-diamidino-2-phenylindole (DAPI) (Vector Labs). The cells were visualized using an epifluorescence microscope (Nikon ECLIPSE 50i). For qualitative analysis three areas were randomly chosen for each line. The signals in the images were quantified using ImageJ64 software by calculating the percentage of cellular lipids per cell to obtain the total surface area of LipidTOX-positive droplets per number of nuclei in one field.

Ca^2+^-containing cells in osteogenic cultures were stained with Alizarin red, whereas the presence of cartilage-related glycosaminoglycans in chondrogenic cultures was detected with Alcian blue staining as described^1^.

### Quantitative RT-PCR

Real-time quantitative PCR (qPCR) was performed with the Roche LightCycler 480 RT PCR Instrument using SYBR Green Mastermix (Roche). Data were collected and analyzed using the comparative threshold cycle method with TATA-box binding protein (*TBP*) and glucuronidase β (GUSB) as reference genes. Mean ± SD was calculated and statistical analysis was performed using the Prism curve-fitting program (GraphPad Prism, version 6.01). Primers pairs for target genes used were: *FABP4*, forward 5′-AGCACCATAACCTTAGATGGG-3 and reverse 5′-CGTGGAAGTGACGCCTTTCA-3′; *COL11A1* forward 5′-CCAGCGTCTGTTGGTTCAGT-3 and reverse 5′-CAGCTTCCCCTTTCTCTCCT-3′; *RUNX2* forward 5′-TTACTTACACCCCGCCAGTC-3′ and reverse 5′-TATGGAGTGCTGCTGGTCTG-3′; *PNPLA7* forward 5′-CACTCTTGGGGACTGTGGTT-3′ and reverse 5′-CGGCCGTAAAACATCACTTT-3′; *TBP* forward 5′-CAGCGTGACTGTGAGTTGCT-3′ and reverse 5′-TGGTTCATGGGGAAAAACAT-3′; *GUSB* forward 5′-AAACGATTGCAGGGTTTCAC-3′ and reverse 5′-CTCTCGTCGGTGACTGTTCA-3′.

### Immunosuppressive assay

The cells were cultured in StemGro, a xeno-free, chemically defined medium (Corning, USA), until they reached 80–90% confluence. The cells were harvested with TrypLE and counted by haemocytometer and evaluated for their viability with Trypan Blue. For the proliferation assay, we used 1 × 10^6^ cells/ml of RPMI medium supplemented with 10% foetal calf serum, 10,000 U/ml penicillin and 10 mg/ml streptomycin (Life Technologies). Human PBMCs were isolated from leukocyte cones purchased from the National Blood Service, UK. Leukocyte cones were diluted 1:1 with PBS and layered on Histopaque (Sigma-Aldrich) for density gradient separation. The proliferation assay was carried out in a 96-well plate (Costar) in a total volume of 200 µl per well. MSCs were plated overnight at serial dilutions. PBMCs, 5 × 10^5^/well, stained in 1 µM violet dye from CellTrace cell proliferation kit (Life Technologies) and stimulated with 5 µg/ml of PHA (Sigma-Aldrich) were added to MSCs to obtain co-cultures with increasing MSC/PBMC ratios from 1:5 to 1:80. BM MSCs were run in parallel as a reference. Cultures of stimulated PBMCs without MSCs were used as a positive control, while non-stimulated PBMCs were used as a negative control. Cells were incubated in 5% CO_2_ at 37 °C for 72 h. The cells were then collected, washed once with PBS, and PBMC proliferation was assessed based on the position and shape of the peak of the cells labelled with the violet dye. Non-stimulated PBMCs were used as guidance for gating in terms of proliferation. In stained cells, the position of the peak increased with increasing dilution. The relative proliferation at each MSC/PBMC ratio was then calculated as a percentage of the proliferation of the positive control by the following formula: A/B × 100, where A is the proliferation at one specific MSC/PBMS ratio and B is the proliferation of the positive control. The percentage of inhibition was then calculated by subtracting the percentage of proliferation from 100 (maximum of inhibition).

### DNA methylation

For a comparative analysis of DNA methylation, we directly sorted CD73^+^CD90^+^CD105^+^ WJ MSCs from the first passage into lysis buffer. Genomic DNA was extracted from triple positive MSCs (CD73^+^, CD90^+^, CD105^+^) using QIAamp DNA Mini Kit (Qiagen). The DNA was quantified using Qubit™ (Thermo Fisher). Five hundred ng of genomic DNA was subjected to bisulfite conversion using the EZ Zymo DNA Kit (Zymo Research) according to the manufacturer’s recommendations for the 450k array. Methylation levels at >480,000 CpG sites throughout the genome were measured using the Infinium Human DNA Methylation 450k BeadChip microarray(Illumina) in triplicate, each array representing an independent bisulfite conversion of a donor sample; performed at the Genomics Facility of the Biomedical Research Centre at the Guy’s and St Thomas’ National Health Service Foundation Trust and King’s College London.

GenoSplice Technology performed quality control, processing, and further analyses of the data. The data were normalised to adjust for assay type II bias using the Beta-MIxture Quantile dilation (BMIQ) method^50^ from the Chip Analysis Methylation Pipeline (ChAMP) R package^51^. The 450 K array contains 65 single nucleotide polymorphism (SNP) probes that do not interrogate methylation, and these were filtered out. In addition, probes having a detection p-value above 0.01 were filtered out, and 1921 probes were thus removed from analysis. The remaining 483591 probes were analysed. The CpGassoc R package^52^ and Methlab software^53^ were used for differential methylation analysis together with the HumanMethylation450 v1.2 Manifest File containing CpG annotations. T-statistics were calculated using logit transformed beta values, which can help stabilise the variance^54^. The logit transform, log(beta/(1-beta)) is equivalent to the log ratio of methylated to unmethylated signal. Multiple testing was performed using the q-value false discovery rate (FDR) method^55^. The genes with at least one CpG site showing a methylation difference ≥0.20 in 4 or more obese donors were further filtered based on the list of polymorphic CpG sites^24^ and segmental duplications^25^.

The methylation data have been deposited in the Gene Expression Omnibus (GEO) under accession number GSE74167.

### TaqMan® Copy Number Assays

TaqMan Copy Number Assays probes and primers specifically designed to target *DCAF6* within intron 17 (essay ID: Hs03384064_cn; Nucleotide sequence variation: nsv946487) and *ZNF714* within intron 2 (essay ID: Hs07155647_cn; Nucleotide sequence variation: nsv960811) were used to analyse a panel of genomic DNA samples isolated from WJ MSC of non-obese and obese donors. All probes and primers including TaqMan Copy Reference Assay RNase P (two primers and a probe) and TaqMan Genotyping Master Mix were purchased from ThermoFisher Scientific.

### Whole Genome Gene-Expression array

The HumanHT-12 Expression BeadChip whole-genome, gene-expression direct hybridization assay system (Illumina) was used at the Genomics Facility of the Biomedical Research Centre at the Guy’s and St Thomas’ National Health Service Foundation Trust and King’s College London. as described^56^. GenoSplice Technology performed quality control, processing, and further analyses of the data. The expression data are now publicly available on GEO under the GSE107214 accession number.

### Western blotting

Frozen cell pellets lysed in RIPA buffer (Pierce), 1 × 10^6^ cells/400 µl, supplemented with protease inhibitor cocktail cOmplete (Roche) were homogenized by passing 10x through 29 G needle and sonicated 2 × 10 sec with sonic dismembranator. Fifteen µl of lysate were mixed with 5 µl of LDS Sample Buffer (Novex® NuPAGE®, Life Technologies), boiled for 5 min, chilled on ice, spun briefly in a table top centrifuge and loaded 15 µl/well of 15-well 4–12% SDS polyacrylamide gel (Novex, NuPAGE, Life Technologies). The proteins were separated in MOPS SDS Running Buffer and transfered from the gel to the 0.2 µm pore size Nitrocellulose membrane (Novex, Life Technologies). The membrane was washed in TBST buffer for 10 min and blocked for 20 min in 5% Bovine Serum Albumin (Sigma-Aldrich, Cat. No. A2153)/TBST. The membrane was then incubated with rabbit anti-PNPLA7 antibody (Sigma, Cat. No. HPA009130) at dilution 1:1000 (20 ng/ml)/TBST on a rocking platform at room temperature for 1 h. The membrane was washed 3 × 10 min in TBST and then incubated with HRP-conjugated donkey anti-rabbit IgG (H + L) cross absorbed secondary antibody (Thermo Scientific) at dilution 1:2500 (20 ng/ml) on a rocking platform at room temperature for 30 min. The membrane was washed 3 × 10 min in TBST. The protein was detected with an enhanced chemiluminsicent Pierce® ECL Western Blotting Substrate (Thermo Scientific). The images were detected, recorded and analysed using Intelligent Dark Box LAS-3000 (FUJIFILM)

The membrane was washed in TBST for 10 min, stripped in Restore™ Western Blot Stripping Buffer (Thermo Scientific) on a rocking platform at room temperature for 15 min and washed again for 10 min in TBST. Membrane was then incubated with a loading control HRP-conjugated mouse anti-actin antibody BA3R (Thermo Scientific) for 1 h, washed 3 × 10 min in TBST and exposed to Pierce® ECL Western Blotting Substrate. The images were detected, recorded and analysed using Intelligent Dark Box LAS-3000.

## Electronic supplementary material

supplementary Information
