# Supplementary material for: Effects of maternal obesity on Wharton’s Jelly mesenchymal stromal cells
Source: Sci Rep. 2017 Dec 14;7:17595. doi: 10.1038/s41598-017-18034-1 (PMC5730612; doi:10.1038/s41598-017-18034-1)
Supplement: Supplementary file 1 — supplementary Information [file 41598_2017_18034_MOESM1_ESM.pdf]

Supplementary Information

Effects of maternal obesity on Wharton's Jelly mesenchymal stromal cells

Heba Badraiq[1], Aleksandra Cvoro[2], Antonio Galleu[3], Marisa Simon[2], Cristian Miere[1], Carl Hobbs[4], Reiner Schulz[5], Richard Siow[6], Francesco Dazzi[3], Dusko Ilic[1]

[1]Stem Cell Laboratory, Assisted Conception Unit, Division of Women's Health, Women's Health Academic Centre, Faculty of Life Sciences & Medicine, King's College London, London, UK.

[2]Genomic Medicine, Houston Methodist Research Institute, Houston, Texas, USA.

[3]Department of Haemato-Oncology, Rayne Institute, Faculty of Life Sciences & Medicine, King's College London, London, UK.

[4]Histology Laboratory, Wolfson Centre for Age-Related Diseases, Faculty of Life Sciences & Medicine, King's College London, London, UK.

[5]Department of Medical and Molecular Genetics, Division of Genetics and Molecular Medicine, and [6]Cardiovascular Division, Faculty of Life Sciences & Medicine, King's College London, London, UK.

Correspondence: Dusko Ilic, E-mail: dusko.ilic@kcl.ac.uk

Non-obese				Obese			
DONOR	AGE	BMI	ETHNICITY	DONOR	AGE	BMI	ETHNICITY
1	43	24	Caucasian	1	39	36	Caucasian
2	35	21	Caucasian	2	35	30	Caucasian
3	36	22	Caucasian	3	34	39	Caucasian
4	35	21	Caucasian	4	34	30	African black
5	38	25	Caucasian	5	38	45	African black
6	39	23	Caucasian	6	28	41	Caribbean black
7	39	23	Caucasian	7	35	35	Caribbean black

Figure S1. Donor information

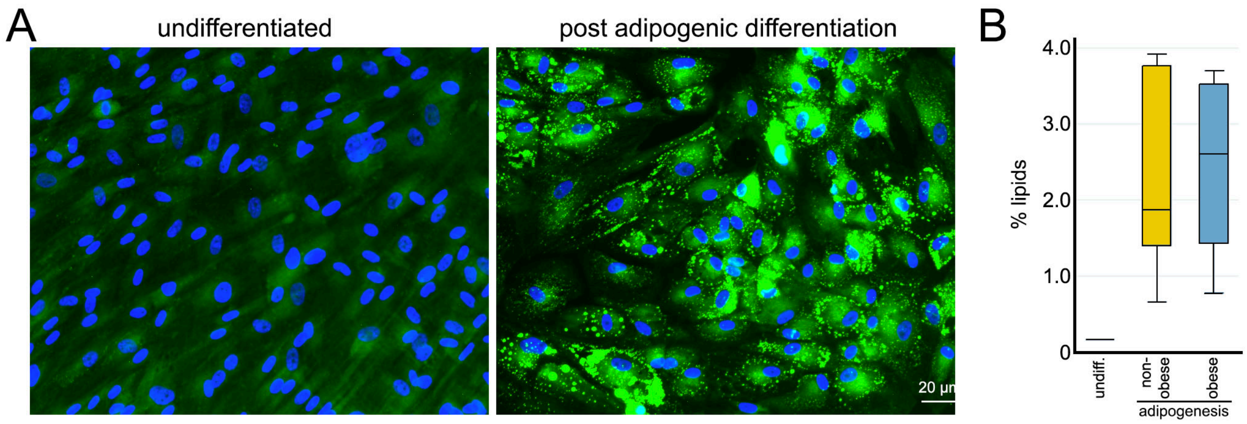

Figure S2. Propensity towards adipogenic differentiation

(A) Intracellular lipid droplets visualized with a fluorescent LipidTOX™ Green in WJ MSC from non-obese donor O1 following proadipogenic differentiation. Undifferentiated cells exposed to the fluorescent dye were used as a negative control to determine a level of background fluorescence.

(B) Quantitative analysis of lipid content per cell did not find any significant difference between samples isolated from non-obese (2.39 ± 1.35%, n=7) and obese donors (2.28 ± 1.10%, n=7).

A

Channel	Intensity	Expected Value	Average values	Results
Green	DNP high	<1000	70	Passed
Green	DNP background	<1000	76	Passed
Green	Biotin (high)	>3000	10328	Passed
Green	Biotin (bkgd)	<1000	40	Passed
Red	DNP high	>3000	6031	Passed
Red	DNP background	<1000	55	Passed
Red	Biotin (high)	<1000	112	Passed
Red	Biotin (bkgd)	<1000	48	Passed

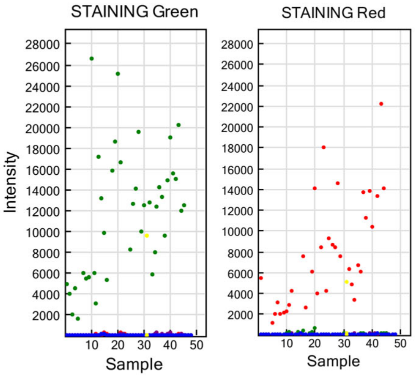

B

Channel	Intensity	Expected Value	Average Value	Results
Green	A	<2000	545	Passed
Green	T	<2000	472	Passed
Green	C	>20000	30512	Passed
Green	G	>20000	29480	Passed
Red	A	>20000	24115	Passed
Red	T	>20000	26279	Passed
Red	C	<2000	1762	Passed
Red	G	<2000	1596	Passed

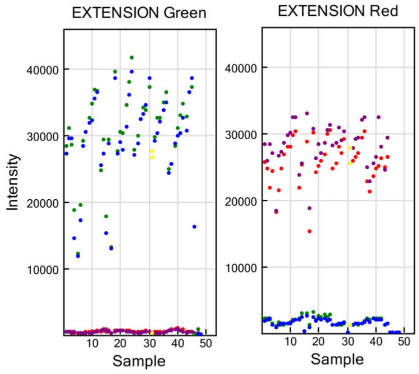

C

Channel	Nucleotide	Expected Value	Average value	Results
Red	A	>5000	9066	Passed
Red	T	>5000	11410	Passed
Red	C	<2000	525	Passed
Red	G	<2000	688	Passed
Green	A	<2000	828	Passed
Green	T	<2000	971	Passed
Green	C	>5000	13087	Passed
Green	G	>5000	9153	Passed

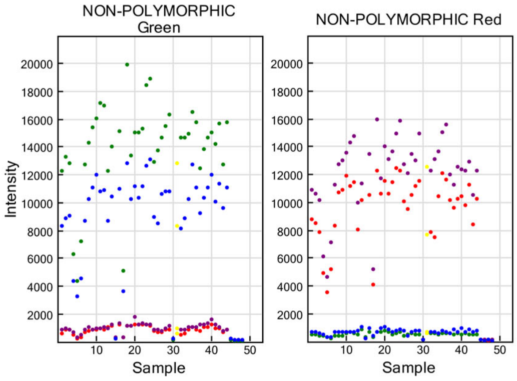

D

Channel	Target Removal Control	Expected Value	Average value	Results
Green	1	<1200	355	Passed
Green	2	<1200	485	Passed

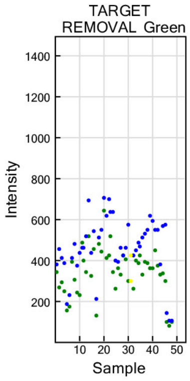

E

Channel	Hyb control concentration	Expected Value	Average value	Results
Green	Low	<10000	8446	Passed
Green	Medium	<20000	16436	Passed
Green	High	>20000	27350	Passed

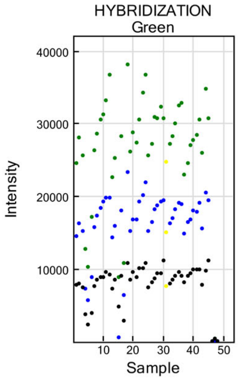

F

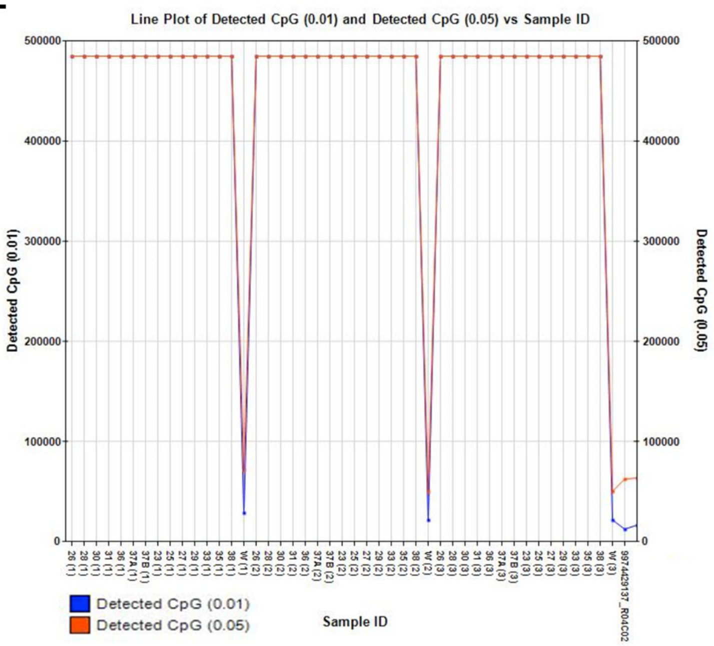

G

Channel	Probe	Expected Value	Average value	Results
Green	C1	>5000	11113	Passed
Green	C2	>5000	11519	Passed
Green	C3	>5000	14438	Passed
Red	C4	>5000	11475	Passed
Red	C5	>5000	8119	Passed
Red	C6	>5000	10924	Passed
Green	U1 to U6	<1000	498	Passed
Red	U1 to U6	<1000	574	Passed

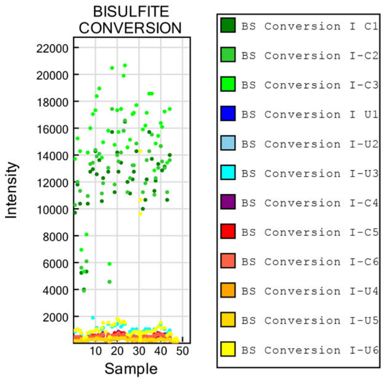

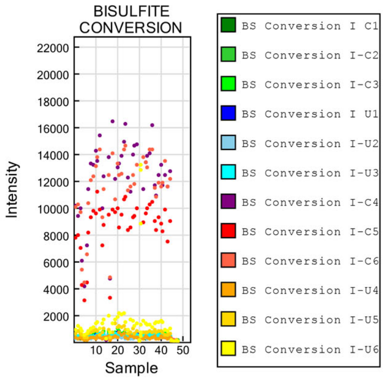

H

Channel	Probe	Expected Value	Average value	Result
Green	1	<3000	1628	Passed
Green	2	<3000	594	Passed
Green	3	<3000	1442	Passed
Green	4	<3000	540	Passed
Red	1	>5000	10278	Passed
Red	2	>5000	10747	Passed
Red	3	>5000	7162	Passed
Red	4	>5000	9796	Passed

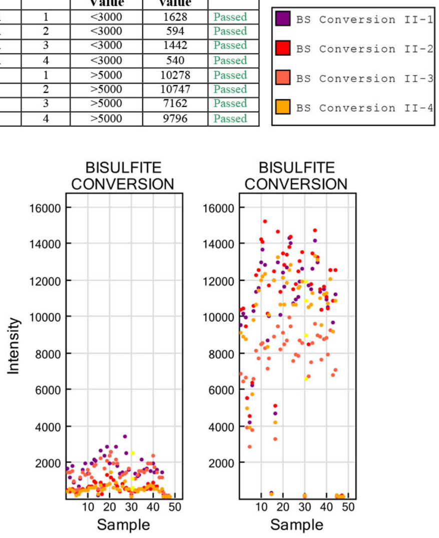

I

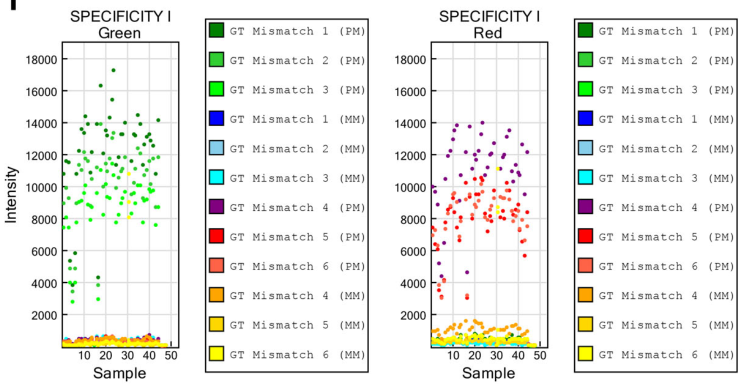

J

Channel	Probe	Expected Value	Average value	Results
Green	1	<3000	482	Passed
Green	2	<3000	426	Passed
Green	3	<3000	385	Passed
Red	1	>5000	10006	Passed
Red	2	>5000	7463	Passed
Red	3	>5000	8951	Passed

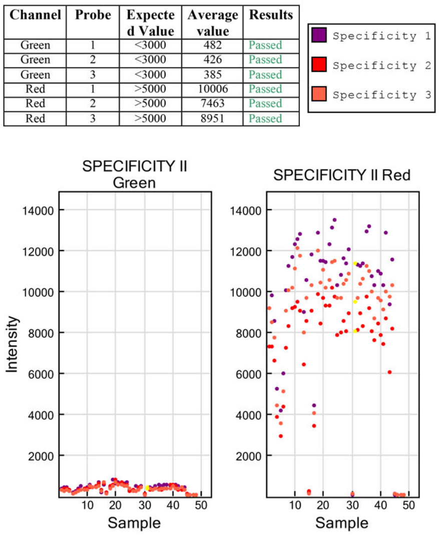

K

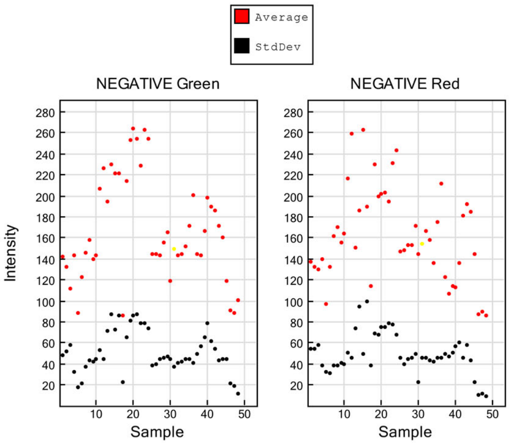

Figure S3. The DNA methylation array passed all quality control parameters examined.

(A) Staining control: Measures the efficiency of the staining step. It is independent of other sample independent controls and data is monitored in both the green and red channel of the iscan during scanning.

(B) Extension control: Tests the efficiency of the single base extension procedure of nucleotides using a hairpin probe. A and T are monitored in the red channel and C and G are monitored in the green channel.

(C) Non-Polymorphic Controls: test the overall performance of the assay, from amplification to detection, by querying a particular base in a non-polymorphic region of the human genome. Non-polymorphic Controls are used to compare assay performance across different samples. A and T are monitored in the red channel, and C and G in the green channel.

(D) Target removal control: Tests the efficiency of the stripping step of excess DNA after the single base extension and is tested only in the green channel.

(E) Hybridisation control: Measures the performance of the Infinium array using synthetic targets to probes on the array with a perfect match, these are tested at three different concentrations, high (5 pM), medium (1 pM) and low (0.2 pM) concentrations. The hybridisation control is only tested for in the green channel.

(F) Detected CpG sites: shows plot of detected CpG sites at 0.01 and 0.05 level.

(G) Bisulfite conversion control 1: Infinium 1 probe design and allele specific single base extension are used to monitor the success of the bisulfite conversion. If the bisulfite conversion reaction was successful the C (converted) probes will match the converted sequence and extend if the bisulfite conversion was not successful then the unconverted (U) probes will get extended.

(H) Bisulfite Conversion control 2: Infinium II probe design and single base extension to monitor the success of the bisulfite conversion, if the conversion is successful the A base will get

incorporated and the probe will have intensity in the red channel, if the sample has any unconverted DNA the G base will get incorporated and the probe will have elevated signal in the green channel.

(I) Specificity Control 1: are designed to monitor the allele specific extension for infinium I probes and uses G/T mismatch controls to check for nonspecific detection of methylation signal over unmethylated background. PM controls correspond to A/T perfect match and should give high signal, MM controls correspond to G/T mismatch controls.

(J) Specificity control 2: are designed to measure the specificity of infinium II probes during single base extension and highlights any non-specific detection of methylation signal over unmethylated background. Infinium II probes incorporates the A base across the non-polymorphic T and show high signal in the red channel and if the incorporation is non-specific the probe will have a high signal in the green channel.

(K) Negative Control - Average and StdDev values should have intensity lower than 600.

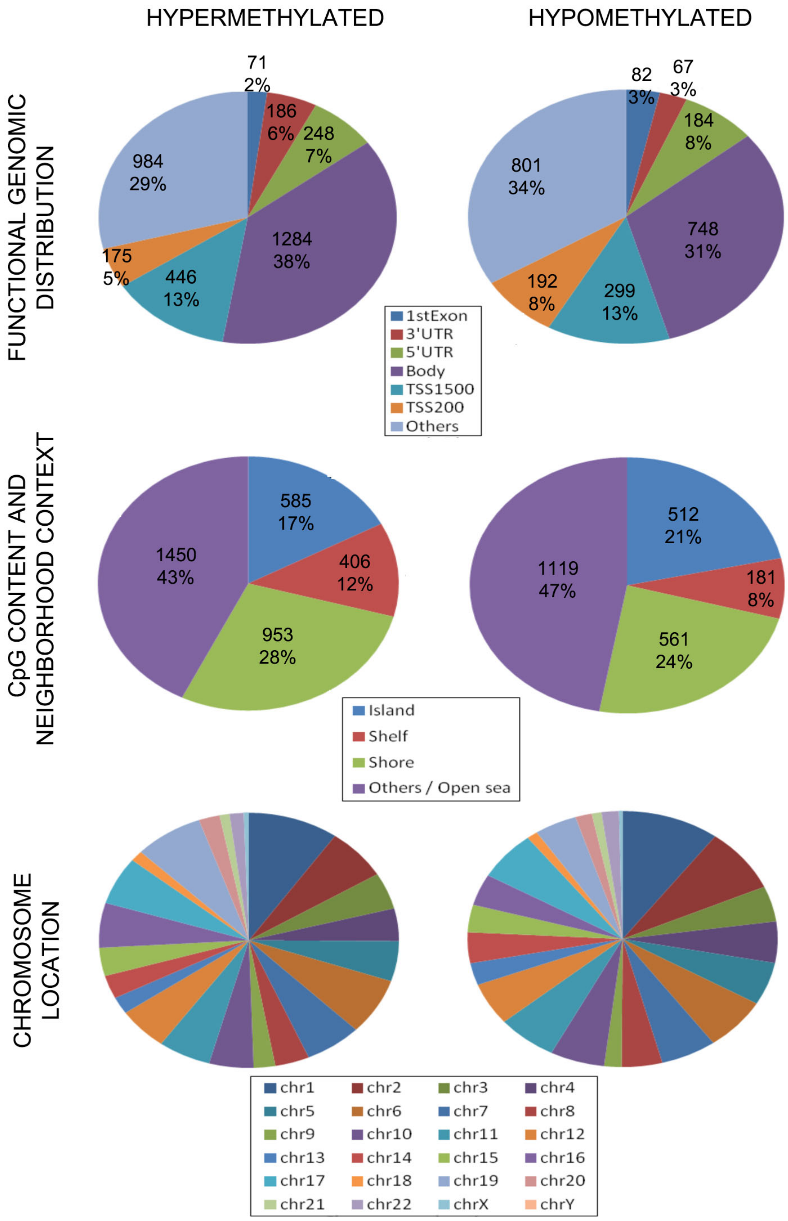

Figure S4. List and genomic distribution of genes with significant methylation differences

(A) Functional genomic distribution of significantly hyper- and hypomethylated CpG sites was quite similar. CpG content and neighbourhood context did not show much difference either. Altered methylation state was spread across all chromosomes, with chromosomes 1, 2 and 6 harbouring the most hyper- and hypomethylated CpG sites.

		Not significant sites (less than 4 donors with methylation difference ≥ 0,20)		Significant sites (4 or more donors with methylation difference ≥ 0,20)		
Gene Name	Number of CpG sites	Number of sites	Number of sites present in polymorphic CpG list	Number of sites	Number of sites present in polymorphic CpG list	All significant site in polymorphic CpG list?
ADAMTS16	14	13	3	1	1	Yes
AGPAT9	2	1	0	1	1	Yes
AHRR	83	82	23	1	0	No
ARHGAP22	28	27	3	1	1	Yes
ARHGEF10	81	80	19	1	0	No
BRDT	4	3	0	1	1	Yes
C14orf119	3	2	0	1	1	Yes
C2orf69	4	3	1	1	0	No
C8orf12	10	9	1	1	1	Yes
CARS2	24	23	5	1	1	Yes
CDH4	70	69	13	1	1	Yes
CDKL1	2	1	0	1	1	Yes
CHST11	16	15	3	1	0	No
CLIP2	20	19	4	1	1	Yes
CTBP1	35	34	8	1	1	Yes
CYP2E1	17	16	1	1	0	No
DCAF11	6	5	1	1	1	Yes
DCAF6	1	0	0	1	0	No
DENND3	55	54	11	1	1	Yes
DOCK2	19	18	4	1	1	Yes
EYA4	36	35	4	1	0	No
GALNT9	116	115	30	1	1	Yes
GHRL	7	6	2	1	1	Yes
GPR37	7	6	1	1	1	Yes
GRID2	20	19	3	1	1	Yes
HLA-DPA1	24	23	10	1	1	Yes
HOOK2	14	13	2	1	0	No
IGSF21	36	35	5	1	1	Yes
ITGB2	21	20	3	1	1	Yes
KCNS3	10	9	1	1	1	Yes
KCTD5	16	15	2	1	1	Yes
LAMB1	11	10	2	1	1	Yes
LILRA6	3	2	0	1	1	Yes
MAD1L1	305	303	55	2	1	No
MTRR	3	2	1	1	0	No
MYO10	32	31	9	1	1	Yes
NDUFS6	20	19	4	1	1	Yes
NEK9	8	7	2	1	1	Yes
NPSR1	4	3	0	1	1	Yes
NRGN	7	6	0	1	1	Yes
NTM	87	86	25	1	1	Yes
OTOF	11	10	0	1	1	Yes
PDHA2	3	2	1	1	1	Yes
PNPLA7	20	19	4	1	0	No
PTGFRN	13	12	2	1	1	Yes
RADIL	41	40	10	1	1	Yes
RPL13AP3	5	4	0	1	1	Yes
SDK1	113	112	29	1	0	No
SEPT8	11	10	1	1	0	No
SERPINE2	12	11	2	1	0	No
SIM2	39	38	2	1	0	No
SLC23A2	9	8	0	1	1	Yes
STT3A	5	4	1	1	1	Yes
TGFBR3	20	19	4	1	1	Yes
THUMPD1	6	5	1	1	1	Yes
TM2D2	2	1	0	1	1	Yes
TMEM44	16	15	5	1	0	No
TNS1	25	24	6	1	1	Yes
TOP1MT	17	16	4	1	1	Yes
TRIM10	36	32	3	4	1	No
TRPC5	5	4	0	1	1	Yes
WDR27	37	36	4	1	1	Yes
ZDHHC14	38	37	6	1	1	Yes
ZNF117	5	4	4	1	1	Yes
ZNF670	5	4	0	1	1	Yes
ZNF714	5	0	0	5	0	No
ZNF783	12	11	3	1	1	Yes

(B) List of the genes with significantly different methylation of at least one CpG site in four or more donors.

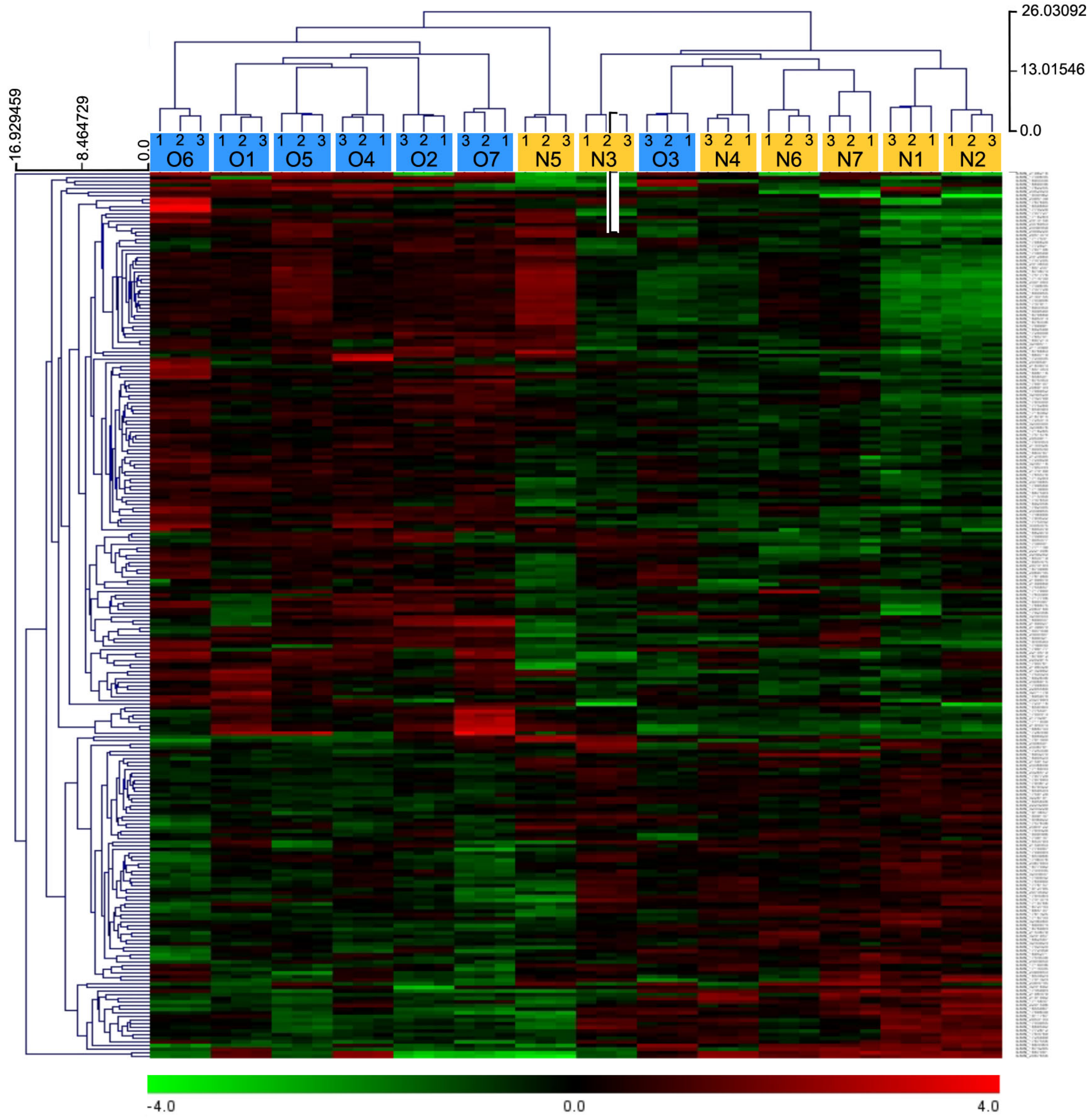

Figure 5. Heat map and a list of the genes with a significantly different transcriptome level

(A) Heat map shows that samples for all obese donors cluster together except one (O3).

Probe_Id	Symbol	FC	P.Value
ILMN_2186216	GOLPH4	5.75	4.85E-05
ILMN_1693338	CYP1B1	4.26	8.04E-10
ILMN_1830462	XYLT1	3.88	6.22E-05
ILMN_1726448	MMP1	3.20	2.54E-04
ILMN_1669046	FOXQ1	3.18	1.24E-04
ILMN_2210519	HAPLN1	3.02	2.60E-06
ILMN_1707727	ANGPTL4	2.66	3.79E-05
ILMN_1770228	KRT34	2.60	3.58E-04
ILMN_2352023	RIPK5	2.57	1.99E-05
ILMN_1716264	ANKRD1	2.57	1.11E-04
ILMN_1782305	NR4A2	2.55	6.51E-08
ILMN_1730645	TMEFF2	2.43	2.63E-03
ILMN_2339955	NR4A2	2.40	2.64E-10
ILMN_1678812	HAPLN1	2.36	1.03E-04
ILMN_1666733	IL8	2.35	2.79E-03
ILMN_2184373	IL8	2.24	2.69E-03
ILMN_2113490	NTN4	2.22	5.43E-05
ILMN_1658483	IL1A	2.20	1.19E-06
ILMN_1775042	WDR69	2.15	1.41E-05
ILMN_1765578	TIPARP	2.15	1.11E-13
ILMN_1799569	LOC440895	2.06	3.71E-08
ILMN_1844593	---	2.05	1.48E-04
ILMN_2374865	ATF3	2.05	1.71E-07
ILMN_1798177	CHURC1	2.03	4.48E-04
ILMN_1658660	ACTC1	2.01	1.53E-02
ILMN_1676663	TNFRSF11B	2.01	6.30E-03
ILMN_1762255	GSTM1	2.01	3.10E-02
ILMN_1682636	CXCL2	1.98	1.41E-05
ILMN_1720829	ZFP36	1.97	3.20E-09
ILMN_1711838	SLC25A24	1.95	1.06E-03
ILMN_3271179	LOC10012877	1.95	2.42E-05
ILMN_2124585	GREM1	1.94	5.05E-07
ILMN_2139970	ALDH1A3	1.94	2.51E-06
ILMN_2132982	IGFBP5	1.93	2.57E-03
ILMN_1723116	AMFR	1.93	2.37E-02
ILMN_3305475	LOC729708	1.91	5.11E-04
ILMN_1690397	DYNC1I1	1.91	1.73E-05
ILMN_2395139	SERPINB7	1.90	1.79E-02
ILMN_1807439	ALDH1A3	1.87	6.14E-05
ILMN_1709800	POMZP3	1.87	8.23E-04
ILMN_1710284	HES1	1.87	2.36E-07
ILMN_1723035	OLR1	1.86	7.72E-03
ILMN_1751776	CKAP2L	1.86	8.47E-03
ILMN_1748840	CALB2	1.86	3.23E-03
ILMN_1775501	IL1B	1.84	3.71E-03
ILMN_2063168	MALL	1.83	1.58E-03
ILMN_1747650	BMP6	1.82	2.74E-06
ILMN_1787186	NOV	1.82	3.00E-04
ILMN_2400407	CNTN1	1.82	3.55E-03
ILMN_1699421	ANXA10	1.81	1.13E-03
ILMN_2174189	DKK1	1.81	1.73E-06
ILMN_1767685	SERPINB7	1.81	2.39E-02
ILMN_1651354	SPP1	1.80	1.35E-04
ILMN_1686116	THBS1	1.80	2.85E-04
ILMN_1805377	ZP3	1.78	3.75E-03
ILMN_1775268	HECW2	1.78	2.19E-05
ILMN_1660718	GABBR2	1.77	1.39E-02
ILMN_1716382	LOC387882	1.77	7.75E-06
ILMN_1746888	PCOLCE2	1.75	6.55E-05
ILMN_1725314	GBP3	1.75	3.07E-07
ILMN_1798952	KDELR3	1.75	4.36E-06
ILMN_1714880	LIMS3	1.74	1.66E-08
ILMN_1737205	MCM4	1.74	2.55E-04
ILMN_1786628	LOC653355	1.74	1.60E-04
ILMN_2249282	TIPRL	1.73	3.26E-05
ILMN_2376953	KCNK2	1.72	2.58E-04
ILMN_1728009	TMEM171	1.72	1.44E-03
ILMN_1766675	CDH6	1.72	7.14E-03
ILMN_1790761	POSTN	1.71	2.18E-03
ILMN_1789991	MARCH4	1.70	4.58E-04
ILMN_2196328	POSTN	1.70	7.83E-03
ILMN_2189027	LIPG	1.69	2.01E-02
ILMN_1714730	UBE2C	1.68	2.20E-02
ILMN_3245116	GOLIM4	1.67	9.01E-09
ILMN_1794522	EIF5A	1.67	1.91E-03
ILMN_2349459	BIRC5	1.67	1.76E-02
ILMN_2068104	TFPI2	1.66	1.30E-04
ILMN_1662970	ZP3	1.66	6.11E-03
ILMN_1739645	ANLN	1.66	2.47E-02
ILMN_1695475	SEMA3C	1.65	1.27E-05
ILMN_1685079	TELO2	1.65	1.05E-02

Probe_Id	Symbol	FC	P.Value
ILMN_1750324	IGFBP5	1.64	7.15E-03
ILMN_1656501	DUSP5	1.64	3.25E-03
ILMN_1788107	IL11	1.64	1.28E-06
ILMN_1795344	GOLPH4	1.64	7.65E-08
ILMN_1784300	TUBA4A	1.63	1.91E-04
ILMN_1683450	CDCA5	1.63	1.64E-02
ILMN_1692056	HS3ST3A1	1.63	5.97E-04
ILMN_1675453	HHIP	1.63	2.01E-04
ILMN_1755657	RASIP1	1.63	5.32E-03
ILMN_2221006	RAD21	1.63	3.23E-04
ILMN_1742789	LPXN	1.63	7.30E-04
ILMN_3240520	ELTD1	1.63	7.03E-05
ILMN_1689037	LIPG	1.62	1.41E-02
ILMN_2285568	NAAA	1.62	1.34E-05
ILMN_1653719	ITGBL1	1.62	1.38E-04
ILMN_1651237	CDT1	1.62	1.28E-02
ILMN_1792356	DPYSL4	1.61	1.51E-02
ILMN_1763390	ISL1	1.61	3.00E-03
ILMN_1678669	RRM2	1.61	3.20E-02
ILMN_2163873	FNDC1	1.61	4.03E-03
ILMN_1717541	CNTNAP3	1.61	1.16E-04
ILMN_1667594	KLF10	1.61	3.98E-06
ILMN_1658494	C13orf15	1.61	3.53E-06
ILMN_1717706	PLK2	1.60	6.17E-04
ILMN_1663787	KLK3	1.60	7.20E-05
ILMN_1739001	TACSTD2	1.59	1.22E-03
ILMN_2202915	FAR2	1.59	6.34E-03
ILMN_1761968	PPP1R14A	1.59	2.50E-03
ILMN_2189869	FCF1	1.59	1.64E-03
ILMN_1747911	CDC2	1.59	2.19E-02
ILMN_1703906	HJURP	1.58	2.47E-02
ILMN_1771149	MRPL19	1.58	4.83E-04
ILMN_2301083	UBE2C	1.58	4.77E-02
ILMN_3240433	GSTT2B	1.58	2.16E-02
ILMN_1687213	C8orf13	1.57	3.28E-03
ILMN_2327994	AZIN1	1.57	4.11E-02
ILMN_1765701	LOC399942	1.56	1.73E-03
ILMN_1673673	PBK	1.56	4.90E-02
ILMN_2409220	HMMR	1.56	4.40E-02
ILMN_2413158	PODXL	1.56	4.45E-02
ILMN_3248511	FAM167A	1.56	4.31E-03
ILMN_2412860	MCM4	1.55	2.77E-03
ILMN_2051373	NEK2	1.55	3.56E-02
ILMN_2144426	HIST2H2AA3	1.55	1.35E-05
ILMN_2173291	CYP4B1	1.55	8.16E-03
ILMN_1676336	AADACL1	1.55	2.57E-02
ILMN_1716265	PGM2L1	1.55	7.77E-08
ILMN_1800540	CD55	1.55	7.22E-06
ILMN_1784454	ITGB1	1.55	1.77E-06
ILMN_1809590	GINS2	1.54	3.12E-02
ILMN_2073184	S1PR5	1.54	2.47E-04
ILMN_1680955	AURKA	1.54	2.72E-02
ILMN_1695414	ASF1B	1.54	3.95E-02
ILMN_1772821	KIAA1671	1.54	1.08E-02
ILMN_1700413	MAFF	1.53	9.33E-03
ILMN_1709683	RASSF2	1.53	9.15E-04
ILMN_1682599	GPRC5A	1.53	4.42E-03
ILMN_2406815	LRRC17	1.53	2.08E-03
ILMN_1715458	PCDH10	1.53	1.00E-04
ILMN_2050911	SLC22A4	1.53	2.69E-10
ILMN_2413650	STIL	1.52	1.03E-02
ILMN_1673998	SSTR1	1.52	1.83E-04
ILMN_2069745	SSTR1	1.52	5.12E-04
ILMN_2189870	FCF1	1.52	1.89E-02
ILMN_1695978	LINGO2	1.51	7.06E-03
ILMN_1738589	MGLL	1.51	8.83E-03
ILMN_1751576	TEK	1.51	4.82E-07
ILMN_2143155	KIF11	1.51	4.14E-02
ILMN_2048591	LRRN3	1.51	1.77E-02
ILMN_2167915	DSEL	1.51	6.19E-04
ILMN_3238676	ULBP2	1.51	2.98E-04
ILMN_3230300	LOC729143	1.50	6.85E-06
ILMN_1737728	CDCA3	1.50	3.86E-02
ILMN_1717990	CALD1	1.50	3.31E-02
ILMN_1667081	CCND2	2.69	1.68E-02
ILMN_1701424	LAMC2	2.63	1.10E-04
ILMN_2196479	XRN2	2.62	5.08E-06
ILMN_1655867	HRASLS	2.54	3.47E-07
ILMN_2067656	CCND2	2.41	4.89E-02
ILMN_1767556	C10orf10	2.35	3.63E-05
ILMN_1745994	GAS7	2.27	6.63E-03

Probe_Id	Symbol	FC	P.Value
ILMN_1664464	PTGDS	2.12	1.21E-02
ILMN_1760990	SH3GL3	2.00	3.02E-05
ILMN_1789639	FMOD	1.97	1.03E-02
ILMN_1781400	SLC7A2	1.94	1.11E-02
ILMN_1686920	CCDC58	1.93	9.93E-03
ILMN_2384745	PSG4	1.89	3.00E-02
ILMN_1761425	OLFML2A	1.87	4.70E-04
ILMN_1653824	LAMC2	1.87	3.57E-04
ILMN_1668592	STON1	1.86	2.73E-03
ILMN_2404850	RPL14	1.84	4.33E-05
ILMN_2053103	SLC40A1	1.82	3.13E-03
ILMN_2181892	BEX2	1.81	7.02E-03
ILMN_2336609	SYTL2	1.79	6.72E-08
ILMN_2406501	SOD2	1.79	2.69E-03
ILMN_1674285	LOC401233	1.78	4.64E-02
ILMN_1676984	DDIT3	1.75	4.37E-06
ILMN_2390853	CTSH	1.74	2.39E-03
ILMN_2367883	GEM	1.69	8.73E-06
ILMN_1665107	ITGB1BP1	1.69	1.06E-03
ILMN_1715647	VANGL2	1.68	1.35E-02
ILMN_1709094	LIFR	1.68	3.94E-05
ILMN_2201596	CYTL1	1.67	6.51E-03
ILMN_1693270	SUSD2	1.67	1.98E-03
ILMN_1740842	SALL2	1.66	6.86E-06
ILMN_1677092	GEM	1.66	2.53E-05
ILMN_1746376	SCARA3	1.65	5.44E-04
ILMN_1809147	FAM118A	1.64	1.22E-03
ILMN_1695606	EFNB3	1.64	2.29E-05
ILMN_1672743	ZNF334	1.64	7.09E-04
ILMN_1797893	PFAAP5	1.63	8.70E-12
ILMN_1703955	FBXO32	1.63	3.98E-03
ILMN_1662587	PNPLA7	1.62	2.37E-06
ILMN_1812795	RUNX1T1	1.62	3.57E-03
ILMN_1758128	CYGB	1.62	1.28E-07
ILMN_1725338	CLDN23	1.62	2.53E-04
ILMN_2073592	CAND2	1.62	6.86E-05
ILMN_2384122	GPR56	1.61	2.19E-04
ILMN_2153679	TBC1D3B	1.61	1.59E-04
ILMN_1718046	ARNT2	1.60	3.92E-04
ILMN_1772612	ANGPTL2	1.60	7.02E-03
ILMN_1797728	HMGCS1	1.59	1.25E-06
ILMN_1811767	INHBE	1.59	2.00E-02
ILMN_2336781	SOD2	1.59	6.45E-03
ILMN_1718766	MT1F	1.59	3.75E-03
ILMN_1695271	RPP25	1.58	1.63E-03
ILMN_1776157	Sep-04	1.58	4.27E-03
ILMN_3233229	SNHG7	1.58	1.37E-07
ILMN_2224290	ZNF322B	1.58	1.57E-05
ILMN_1778087	ANXA8	1.57	1.25E-02
ILMN_1653794	C6orf160	1.57	3.89E-02
ILMN_3226181	NUDT7	1.57	7.04E-06
ILMN_1658504	CHKA	1.57	2.66E-06
ILMN_1714335	RDH10	1.56	2.98E-03
ILMN_2159152	TP53TG3	1.56	2.38E-06
ILMN_1654966	SCARA3	1.56	2.96E-03
ILMN_1772459	RPS23	1.56	1.53E-02
ILMN_1725889	AFF2	1.56	5.29E-03
ILMN_3234837	PKDCC	1.55	2.57E-04
ILMN_3246060	LOC654433	1.55	8.80E-04
ILMN_1731374	CPE	1.55	4.24E-02
ILMN_1678422	DHX58	1.55	3.10E-07
ILMN_1716843	ELOVL2	1.55	3.50E-05
ILMN_3241692	LOC10012966	1.55	2.80E-02
ILMN_3243924	P2RX6	1.55	9.04E-05
ILMN_2159453	STXBP2	1.54	7.07E-03
ILMN_1846922	---	1.54	2.99E-04
ILMN_1809496	COPG2	1.53	1.65E-03
ILMN_1680874	TUBB2B	1.52	2.09E-03
ILMN_1784364	STARD5	1.52	2.54E-02
ILMN_3241957	LOC10013393	1.52	8.07E-06
ILMN_1754538	C10orf58	1.52	7.41E-03
ILMN_1764769	VWA5A	1.52	2.92E-03
ILMN_1738147	NES	1.52	3.29E-04
ILMN_1814657	TFAP4	1.52	7.62E-06
ILMN_1757636	C5orf35	1.52	3.64E-03
ILMN_1716733	MYOM2	1.51	5.71E-04
ILMN_2326512	CASP1	1.51	8.76E-05
ILMN_1702320	JAKMIP2	1.51	1.55E-02
ILMN_1794612	UBA7	1.51	2.07E-08
ILMN_1734445	LOC91461	1.51	9.60E-04
ILMN_1690523	LRRC20	1.50	6.79E-05
ILMN_1784428	MGC57346	1.50	6.29E-04

(B) List of the genes with significantly different transcriptome level between two groups.

Link to KEGG Pathway	Pathway Description (KEGG)	Nb Genes in Pathway	Nb Regulated Genes (Up / Down)	P-Value (All)	P-Value (Up)	P-Value (Down)	Min P-Value
hsa00980	Metabolism of xenobiotics by	60	4 (4/0)	NA	1.75E-02	NA	1.75E-02
hsa04512	ECM-receptor interaction	84	5 (4/1)	3.47E-02	4.18E-02	NA	3.47E-02
hsa04640	Hematopoietic cell lineage	86	4 (4/0)	NA	4.44E-02	NA	4.44E-02

Link to REACTOME Pathway	Pathway Description (REACTOME)	Nb Genes in Pathway	Nb Regulated Genes (Up / Down)	P-Value (All)	P-Value (Up)	P-Value (Down)	Min P-Value
REACT_152	Cell Cycle, Mitotic	304	12 (11/1)	1.03E-03	4.05E-04	NA	4.05E-04
REACT_17015	Metabolism of proteins	217	4 (0/4)	NA	NA	1.61E-02	1.61E-02

Term Type	GO ID with Link	Go Term	Nb Genes in Term	Nb Regulated Genes (Up / Down)	P-Value
cellular_component	GO:0005576	extracellular region	2010	44 (32/12)	1.68E-05
cellular_component	GO:0044421	extracellular region part	960	27 (22/5)	2.47E-05
biological_process	GO:0000280	nuclear division	220	12 (11/1)	5.53E-05
biological_process	GO:0007067	mitosis	220	12 (11/1)	5.53E-05
biological_process	GO:0000087	M phase of mitotic cell cycle	224	12 (11/1)	6.52E-05
biological_process	GO:0048285	organelle fission	229	12 (11/1)	7.93E-05
biological_process	GO:0007049	cell cycle	776	23 (18/5)	1.02E-04
biological_process	GO:0000278	mitotic cell cycle	370	15 (13/2)	1.13E-04
biological_process	GO:0022402	cell cycle process	565	18 (14/4)	3.34E-04
cellular_component	GO:0031012	extracellular matrix	345	13 (10/3)	5.17E-04
biological_process	GO:0042221	response to chemical stimulus	1281	29 (19/10)	8.76E-04
molecular_function	GO:0005102	receptor binding	886	22 (16/6)	1.02E-03
biological_process	GO:0022403	cell cycle phase	414	14 (12/2)	1.15E-03
molecular_function	GO:0005125	cytokine activity	195	9 (9/0)	1.69E-03
biological_process	GO:0000279	M phase	329	12 (11/1)	1.69E-03
molecular_function	GO:0030247	polysaccharide binding	154	8 (6/2)	1.83E-03
molecular_function	GO:0001871	pattern binding	154	8 (6/2)	1.83E-03
biological_process	GO:0007346	regulation of mitotic cell cycle	152	8 (8/0)	2.05E-03
molecular_function	GO:0005515	protein binding	8154	112 (74/38)	2.07E-03
biological_process	GO:0048731	system development	2330	43 (28/15)	2.11E-03
biological_process	GO:0010564	regulation of cell cycle process	114	7 (7/0)	2.19E-03
biological_process	GO:0050793	regulation of developmental process	674	18 (15/3)	2.32E-03
biological_process	GO:0051301	cell division	295	11 (9/2)	2.47E-03
biological_process	GO:0045766	positive regulation of angiogenesis	26	4 (4/0)	3.34E-03
biological_process	GO:0007155	cell adhesion	700	18 (14/4)	3.42E-03
biological_process	GO:0022610	biological adhesion	701	18 (14/4)	3.47E-03
biological_process	GO:0042493	response to drug	216	9 (5/4)	3.89E-03
biological_process	GO:0007275	multicellular organismal development	2865	49 (32/17)	4.29E-03
biological_process	GO:0009991	response to extracellular stimulus	220	9 (6/3)	4.35E-03
cellular_component	GO:0005615	extracellular space	685	17 (14/3)	4.38E-03
biological_process	GO:0009888	tissue development	665	17 (14/3)	4.91E-03
molecular_function	GO:0005539	glycosaminoglycan binding	140	7 (6/1)	5.13E-03
biological_process	GO:0048856	anatomical structure development	2527	44 (28/16)	5.50E-03
biological_process	GO:0051726	regulation of cell cycle	331	11 (10/1)	5.57E-03
biological_process	GO:0045765	regulation of angiogenesis	63	5 (5/0)	6.27E-03
biological_process	GO:0007399	nervous system development	1088	23 (11/12)	8.10E-03
biological_process	GO:0007096	regulation of exit from mitosis	12	3 (3/0)	8.29E-03
biological_process	GO:0031667	response to nutrient levels	197	8 (5/3)	8.44E-03
biological_process	GO:0043933	macromolecular complex subunit organization	710	17 (10/7)	9.00E-03
cellular_component	GO:0000267	cell fraction	1083	22 (12/10)	9.68E-03
cellular_component	GO:0005578	proteinaceous extracellular matrix	320	10 (8/2)	1.00E-02

Figure S6

Putative pathways affected with transcriptome differences between obese and non-obese donors

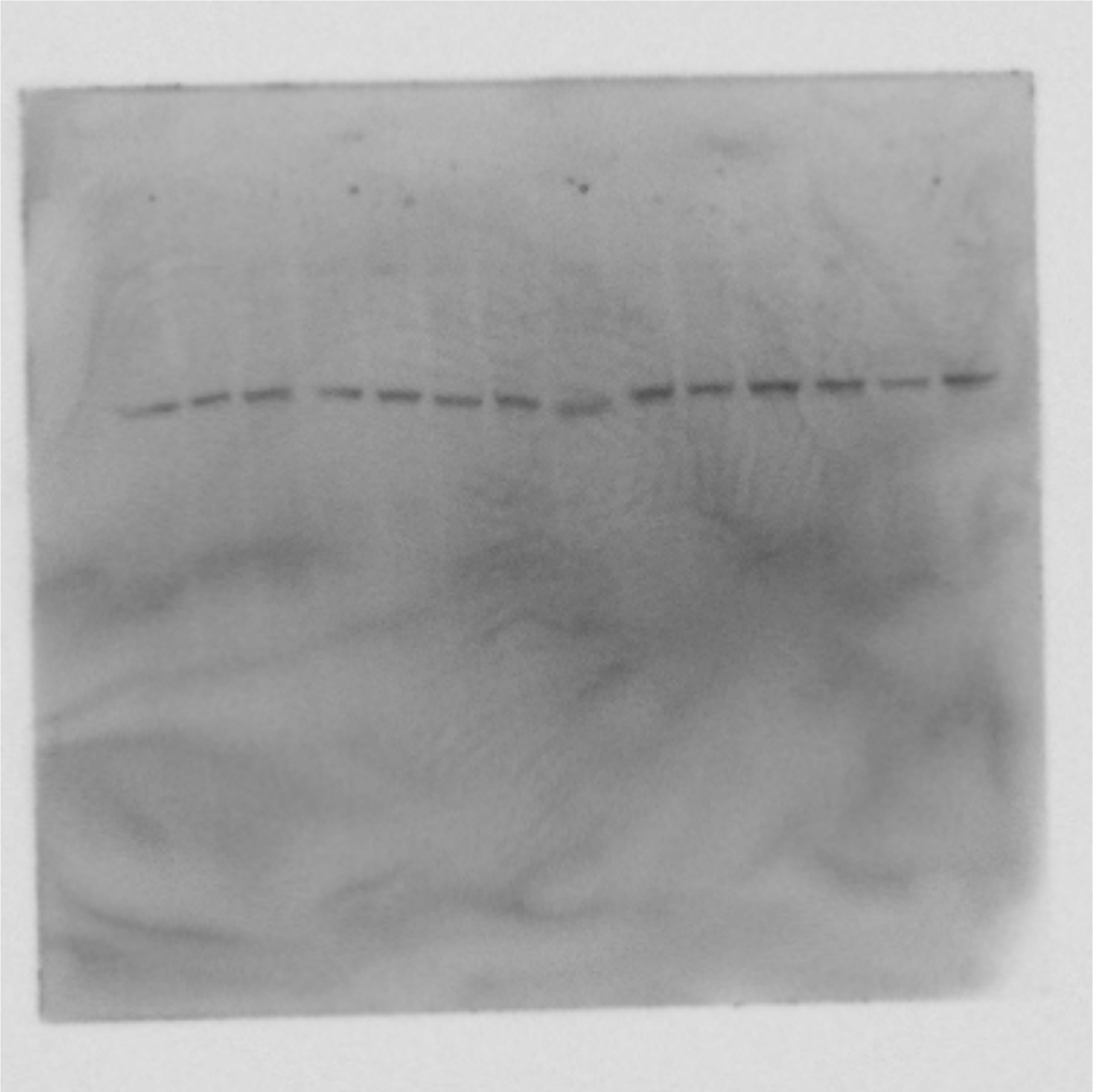

Figure S7

Western blot analysis of PNPLA7 protein levels in samples from non-obese and obese donors – full-length blot (see Figure 6D for annotation).
